# Population Pharmacokinetics and Pharmacodynamics Modeling of Torasemide and Furosemide After Oral Repeated Administration in Healthy Dogs

**DOI:** 10.3389/fvets.2020.00151

**Published:** 2020-04-28

**Authors:** Ludovic Pelligand, Emilie Guillot, Anne Geneteau, Jerome Guyonnet, Reynald Magnier, Jonathan Elliott, Mathieu Peyrou, Matthieu Jacobs

**Affiliations:** ^1^Department of Comparative Biological Sciences, The Royal Veterinary College, London, United Kingdom; ^2^Companion Animal Franchise, Ceva Santé Animale, Libourne, France; ^3^R&D, Ceva Santé Animale, Libourne, France

**Keywords:** loop-diuretic, natriuresis, diuresis, urine, Non Linear Mixed Effect Model (NLME), aldosterone, PK/PD, modeling

## Abstract

Torasemide is a loop diuretic licensed in dogs for cardiogenic pulmonary oedema. The aim of this pharmacokinetic-pharmacodynamic (PK/PD) study was to define an optimally effective dosage regimen based on preclinical data. In a first study, 5 dogs received once-daily oral torasemide (0, 0.1, 0.2, 0.4, 0.8 mg/kg/day) for 14 days. A second study compared once-daily oral torasemide (0, 0.1, 0.2, 0.3, 0.4 mg/kg/day) to twice-daily furosemide (1, 2, 4, 8 mg/kg/day). For all doses of the second study, 11 dogs received a first day of treatment, followed by a 3 day washout and resumed daily treatment for 10 days (until Day 14). Blood and urine were collected to measure urinary torasemide excretion and plasma torasemide concentrations and daily diuresis and natriuresis. Torasemide PK was linear. After rapid absorption (T_max_ 0.5–1 h), 61% of the bioavailable torasemide was eliminated unchanged in urine. Diuresis and natriuresis observed with torasemide were similar to the ones obtained after furosemide (daily dose-ratios: 1/20 to 1/10). The average diuresis increased from baseline (220 ± 53 mL/day for 10 kg dogs) to 730 ±120 mL after the first torasemide administration and up to 1150 ± 252 mL after 10 administrations at the highest dose. At higher doses (≥0.3 mg/kg/day), daily diureses after 10 diuretic treatment-days were higher than Day 1 and variable between dogs; in contrast, diureses remained constant over time and less variable for doses up to 0.2 mg/kg/day. Natriuresis peaked after the first day and decreased dramatically after the 2nd treatment-day then stabilized to a value close to baseline, except for 0.4 mg/kg/day. Urinary torasemide excretion predicted pharmacodynamics better than plasma concentrations. The decrease in natriuresis observed was successfully modeled using a resistance mechanism; this is likely due to a reabsorption of sodium which did not seem however to affect the volume of urine excreted. For a daily target diuresis of 460 mL/dog/day in severe pulmonary oedema (net fluid loss 240 mL/dog/day), a computed dose of 0.26 mg/kg/day (3.5 mg/kg/day furosemide-equivalent) was selected for clinical studies. Due to high inter-individual variability in diureses at doses ≥0.3 mg/kg, higher doses should be limited to 3–5 days to avoid supra-clinical effects in high responders.

## Introduction

Acute congestive heart failure (CHF) in dogs originates either from mitral valve disease or dilated cardiomyopathy (1, 2). The prevalence of these aetiologies and long term-prognosis vary depending on breed. The pathophysiology of CHF is the same across all species with respect to activation of the Renin Angiotensin Aldosterone system (RAAS) and the development of volume overload. Loop diuretics are the cornerstone for the management of acute and chronic decompensated heart failure and associated cardiogenic pulmonary oedema. The goal of loop diuretic treatment of heart failure is to achieve negative salt and water balance for reducing pulmonary oedema (acute decongestion) in the short term and reduce extracellular fluid volume chronically (3). Furosemide has been used historically for 50 years in both human and animals. Furosemide dose was determined empirically though individual dose-titration tailored to each dog, with doses ranging between 5 and 8mg/kg/day for acute decongestion and 1–2 mg/kg/day for maintenance of euvolaemia.

Torasemide is a newly licensed loop diuretic for the treatment of congestive heart failure in dogs, with once-daily administration. It has been licensed in human medicine since 1993, by the FDA. Torasemide and furosemide both inhibit the activity of the Na^+^-K^+^-2Cl^−^ symporter in the thick ascending limb of the loop of Henle by binding to chloride binding site of the symporter from the luminal side; hence the time-course of urinary concentration of loop diuretic is relevant to the activity of the drug (4). Torasemide has several advantages over furosemide. The oral absorption of torasemide is reproducible as its bioavailability is consistently high in fasted and fed states, reaching 98% when fed (5), vs. 77% with furosemide. Although torasemide is partially metabolized in non-active metabolites, the kidney remains the major route of elimination as 60% of torasemide is excteted in urine unchanged, vs. around 20% with furosemide. The filtration of torasemide is limited by its extensive binding to plasma protein in dogs [98.4%, (6)] but is actively transported into the proximal tubule by the organic anion transport system. After repeated administration, diuretic resistance develops with furosemide whereas the diuretic effect is preserved at 14 days with torasemide (7). Finally, although torasemide is not a mineralocorticoid receptor antagonist (8), possible indirect blockade of aldosterone action could have beneficial antifibrotic effect through other anti-remodeling mechanisms (indirect anti-aldosterone effect), explaining a possible long-term survival benefit of torasemide over furosemide in people (9).

The objective of this paper is to propose a data-driven computation of an optimally effective dosage regimen of torasemide for dogs with pulmonary oedema, based on preclinical data. The analysis relied on the evaluation of torasemide effects after repeated oral administrations of different doses administered to healthy dogs, using two approaches: (i) first a comparison of diuretic effect with different doses of torasemide vs. furosemide to establish the dose ratio of torasemide vs. the benchmark loop diuretic, then (ii) applying pharmacokinetic (PK)/pharmacodynamic (PD) modeling methodology to justify a range of clinical doses required to achieve a targeted daily fluid loss. Using this approach in drug development offers the possibility to pool data from different studies to gain integrated information on pharmacokinetics and pharmacodynamics (better estimation of parameters and their individual variability due to complementary datasets) (10).

In the present study, we propose to use a population PK/PD approach to pool torasemide data from two randomized preclinical placebo- and furosemide-controlled cross-over studies.

## Materials and Methods

### Studies Designs and Data Collection

Two studies (Table 1 and Table S1) were performed in accordance to European Directive 2010/63/EU on the protection of animals used for scientific purpose (which intrinsically enforces the 3Rs in its Article 4). Torasemide (C_16_H_20_N_4_O_3_S. Molecular weight: 348.421 g/mol. CAS #: 56211-40-6) was administered as 2, 4, and 8 mg tablets (ISEMID®, Ceva Santé Animale, Libourne, France) and furosemide (C_12_H_11_ClN_2_0_5_S, Molecular weight: 330.75 g/mol, CAS #: 54-31-9) was administered as 10 and 40 mg tablets (Dimazon®, MSD Santé Animale, Beaucouzé, France). All dogs were healthy based on clinical examination by a veterinarian, hematology and biochemistry analyses.

**Table 1:** List of the PK/PD studies with subject demographics, study design and torasemide doses.

**Study**	**Objectives**	**Test product Dosage regimen Route of administration**	**N^**°**^ of dogs weight and age**
Study 1	Pharmacokinetic (PK) and Pharmacodynamic (PD) of torasemide Repeated daily oral doses (14 days) *Pilot study*	5 periods: Placebo, torasemide 4 doses 0.1, 0.2, 0.4, and 0.8 mg/kg once a day*Oral route* Wash-out between periods: 2 weeks	5 healthy male Beagle dogs 1–2 yo (10.1 kg avg.)
Study 2	PK/PD + accumulation after a single, then 3 day-break followed by repeated oral doses (14 days) *GLP study*	9 periods: Placebo, torasemide 4 doses, furosemide 4 doses ISEMID® tablets (torasemide) 0.1, 0.2, 0.3, and 0.4 mg/kg once a day Dimazon® tablets (furosemide) 1, 2, 5, and 8 mg/kg/day in 2 adm. 12 h apart *Oral route* Wash-out between periods: 2 weeks	12 healthy male Beagle dogs 1 yo (10.6 kg avg.)

The first study was a randomized 5-period placebo-controlled crossover study exploring 4 doses of torasemide (0.1, 0.2, 0.4, and 0.8 mg/kg), administered once daily (morning) for 14 days (Table 1). Five male Beagle dogs were included (9.3 to 11.6 kg, 1 to 2.1 year old). Tablets were administered by oral gavage approximately 30 min after the food distribution and flushed with water (3 to 5 mL). Wash-out period was 2 weeks.

Jugular blood samples were collected twice at baseline (once in the morning of days minus 3 and minus 1, before feed intake), on the first day of treatment (before food intake and at 0.25, 0.5, 0.75, 1, 1.5, 2, 4, 6, 8, 10, and 24 h after treatment administration), on Day 2 to 13 (before food intake then 2 h post dosing) and after the last treatment of Day 14 (before food intake then serially up to 72 h after treatment administration), as shown in Table S1. Blood samples were collected into lithium heparin tubes for plasma torasemide measurement and coagulation activator tubes for plasma aldosterone measurement. Samples were centrifuged as soon as possible at 2,500 g for 10 min at 2–8°C, plasma/serum was aliquoted and stored frozen at −80°C pending analysis.

Urine was collected twice over 24 h at baseline (from Day minus 3 to minus 2 and within 24 h prior to drug administration) and over 24 h after treatment on Day 1 and 14 (collection periods: 0–2, 2–4, 4–6, 6–8, 8–10, 10–12, 12–24h). Dogs were encouraged to urinate to make sure they had an empty bladder before putting them in metabolism cages. Urine was collected at 5°C in pre-weighed plastic cooled containers. The containers were weighed and urine specific gravity measured (refractometer) to calculate urine volume. In case of urine absence in the container after each collection interval, appropriate methods were used to collect urine (manual expression of the bladder or catheterization if manual expression was not successful). Catheterization was not used more than twice a day to reduce the risk of iatrogenic infection. Urine aliquots were stored at −80°C for determination of urinary torasemide and sodium concentrations.

The second study was a 9-period placebo-controlled crossover study evaluating the effects of 4 doses of torasemide and furosemide compared to placebo, see Table 1. Another group of 12 adult male Beagle dogs (9.9–11.2 kg) and aged 15 to 19 months was enrolled.

For each period, the dogs received one of the treatments on Day 1 and from Day 5 to 14 (but no treatment from Day 2 to 4, in order to evaluate the pharmacokinetics and pharmacodynamics after the first treatment day). The morning administration occurred about 30 min after the start of food distribution. Torasemide was administered once daily (in the morning) at 0.1, 0.2, 0.3, and 0.4 mg/kg/day and furosemide twice daily (12 h dosing interval) at 0.5, 1, 2.5, and 4 mg/kg/12 h. The washout between periods was 2 weeks.

Blood samples were collected at baseline (once in the morning of days minus 3 and minus 1, before feed intake), on the first day of treatment (before food intake and at 0.25, 0.5, 0.75, 1, 1.5, 2, 4, 6, 8, 12, and 24 h after treatment administration), on Day 12 and 13 (before food intake) and after the last treatment of Day 14 (before food intake then serially up to 96 h after the last administration).

### Torasemide and Biochemistry Analyses

A LC-MS/MS assay for the determination of torasemide was developed and validated in 0.1 mL of dog urine. After the addition of the internal standard torasemide-d6 and 0.2 mL of phosphate buffer solution pH5 (VWR, Fontenay-sous-Bois), torasemide was isolated through solid phase extraction (Waters, Milford). Chromatographic separation was achieved isocratically on a C_18_ column 2.1 × 50 mm, 3.5 μm at 0.15 mL/min. The mobile phase contained 0.1 formic acid in water/acetonitrile (65:35; v/v%). Detection was accomplished using a Sciex API 2000 tandem mass spectrometer in positive ion electrospray SRM mode. The runtime was 5.min. The standard curves, which ranged from 20 to 120,000 ng/mL for torasemide, were fitted to a 1/x^2^ weighted linear regression model. The intra-assay precisions, based on five levels of QC samples (LLOQ, low, medium, high and ULOQ), were within 6.40% CV. The intra-assay precisions, based on four levels of QC samples (low, medium, high and ULOQ), were within 4.75% CV. At the lower limit of quantitation (LLOQ) of 20 ng/mL for torasemide, the inter-assay deviations of the predicted concentrations from the nominal values were within 4.43% for torasemide. The specificity was demonstrated: the response measured in blank samples is <20% of the response measured at the lower limit of quantitation. Stability of torasemide in dog urine was demonstrated for approximately 9 months at −80°C (see method validation data in Table S2).

A LC-MS/MS method was developed and validated for the quantitation of torasemide in 0.1 mL of dog plasma. The method utilized torasemide-d6 as internal standard. After the addition of the internal standard, the samples were processed using crash protein precipitation with methanol. Chromatographic separation was achieved with gradient elution on a C_18_ column 100 × 3 mm, 3.5 μm at 0.4 mL/min. The mobile phase contained water, ethanol and formic acid. Detection was accomplished using a Sciex API 3000 tandem mass spectrometer in positive ion electrospray SRM mode. The standard curves, which ranged from 5 to 4,000 ng/mL, were fitted to a 1/x^2^ weighted linear regression model. The intra-assay precisions, based on five levels of QC samples (LLOQ, low, medium, high and ULOQ), were within 4.88% CV and inter-assay precisions were within 9.04% CV. At the lower limit of quantitation (LLOQ), the inter-assay accuracy were within 1.72%. The specificity of the method was demonstrated. The LLOQ response ratio, when compared to the QC_0_ response ratio, was >5. Torasemide was stable in dog plasma for at least 184 days at −80°C (Table S1).

Urinary sodium was measured by a potentiometric method (KONELAB 60). A ligand-binding assay was validated for the quantitation of aldosterone in dog serum. The aldosterone kit (Demeditec Diagnostics GmbH) was a solid phase enzyme-linked immunosorbent assay (ELISA), based on the principle of competitive binding. The microtiter wells were coated with a polyclonal rabbit antibody directed toward an antigenic site of the aldosterone molecule. Endogenous aldosterone of study samples competed with an aldosterone-horseradish peroxidase conjugate for binding to the coated antibody. After incubation the unbound conjugate was washed off. After addition of the substrate solution, the intensity of color developed was invertedly proportional to the concentration of aldosterone in the sample. The quantification range was 10 to 1,000 pg/mL. The calibration curve was fitted to a 4-parameter logistic regression model. The inter-assay precision was within 35.3% CV and the intra-assay precision was within 35.3% CV. The inter-assay accuracy was within ± 7.3% of the nominal values. Aldosterone in dog serum was stable for 6 h at room temperature, 2 months at −20°C and was also stable for three freeze-thaw cycles.

### Torasemide Population Pharmacokinetic and Pharmacodynamic Analysis

Data from the two studies were pooled; in study 1, blood samples were collected every day with repeated administration of torasemide, whereas study 2 focused on the first and the last dose for the plasma samples.

The population pharmacokinetic—pharmacodynamic analysis was performed using Nonmem® 7.3 software (ICON, USA). All the population PK and PD analyses were performed with the First Order Conditional Estimation (FOCE) method. Data generated from study 1 and 2 were pooled together and analyzed simultaneously. In a previous target animal safety study (in preparation for submission), dilatation of tubules and subcapsular cysts in the renal cortex were observed at the high torasemide dose of 0.8 mg/kg. Therefore, to avoid any bias in the PK/PD analysis due to modification in the site of action, the PK/PD analysis was performed without data from the 0.8 mg/kg dosing period.

#### General Approach to Model Building

**Figure 1:**
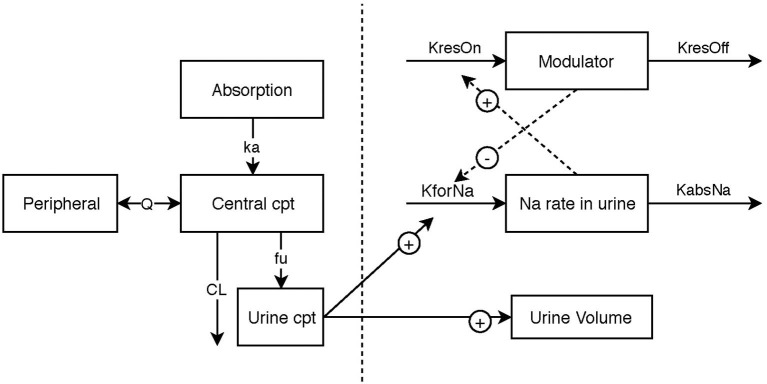
Schematic illustration of the final pharmacokinetic/pharma model, linking urinary torasemide excretion to plasma torasemide concentrations and the two pharmacodynamic submodels for diuresis and natriuresis. The effect of torasemide on diuresis was modeled with a direct response (power model). The natriuresis was modeled with an indirect response model. A modulator inhibiting the formation rate of sodium in urine was included to model reversible resistance to Torasemide (negative feedback loop).

Model building was performed in two steps. The first step was to identify the structural model, which is the model that best described the data in the absence of variability from PK data or from PD data. Once the structural models have been established (Figure 1), the variability of the parameters was assessed for each model.

In a population analysis, there are usually two sources of variability: the inter-individual variability (IIV) on the one hand, and the residual (unexplained) variability, on the other hand (11).

The typical parameters of the population were estimated as well as the IIV (assuming a log-normal distribution). Each parameter was modeled following the usual exponential model.

Hence, the model parameter for the i^*th*^ subject (θ_i_) is written as:

$$\begin{array}{l} \theta_{i}=\theta_{\mu }\;*\;EXP\left(\eta_{i}\right) \end{array}$$

Where θ_i_ is the parameter of the i^th^ subject, θ_μ_ is the population mean of the parameter in question also called typical value and a random parameter eta (η_i_), which is the deviation from the mean for the i^th^ subject. Eta distribution has for mean zero and for variance ω^2^ (i.e., IIV of the parameter).

$$\begin{array}{l} \eta \approx N\left(0,\omega^{2}\right) \end{array}$$

In the present analysis, a diagonal matrix formed by the variance terms was selected (covariances were not estimated). When a log-normal distribution of parameters assumed, the variance estimate is in the log-domain and does not have the same magnitude as the θ values. The following equation converts the variance to a coefficient of variation (CV%) in the original scale (11).

$$\begin{array}{l} \text{CV}\left(\%\right)=100\times \sqrt{\exp\left(\omega^{2}\right)-1} \end{array}$$

Parameter estimation with their associated SE as a measure of the precision of the estimation was based on minimizing an objective function value (OFV), using maximum likelihood estimation. The OFV, expressed as minus twice the log of the likelihood (-2LL), is a single number that provides an overall summary of how closely the model predictions (given a set of parameter values) match the data (maximum likelihood = lowest OFV = best fit). The likelihood ratio test (LRT), was performed to statistically compare different nested models: a reduction of at least 10.83 (corresponding to a *P* < 0.001 for 1 degree of freedom) was required to choose the more complex model.

Shrinkage of random effect toward the typical values can occur when data are not dense enough to estimate the random component. The shrinkage for the etas were estimated as follows (11):

$$\begin{array}{l} shrinkage=1-\frac{SD\left(EBE_{\eta }\right)}{\omega } \end{array}$$

where ω is the estimated variability for the population and SD is the SD of the individual values of the Empirical Bayesian Estimates (EBE) of η. When η shrinkage are high (>30%), plotting individual-predicted concentrations or parameters vs. a covariate (as in diagnostic plots) may be misleading but model comparisons on Objective Value Functions (OVF) and population predictions are unaffected.

The estimation of IIV for certain parameters was decided based on data profiles, precision of parameter estimates in the structural model, η shrinkage and plausibility. The resulting model performance was assessed by the OFV, the goodness of fits plots (DV vs. IPRED, CWRES vs. PRED, Visual predictive check), the plausibility of parameter estimates and their precision, and the residual variability.

For residual variability, the residual model was multiplicative (proportional) plus an additive model of the form:

$$\begin{array}{l} C\left(t\right)=f\left(\theta ,Time\right)\times \left(1+\varepsilon_{1}\right)+\varepsilon_{2} \end{array}$$

with two random effects, ε1 and ε2, as the multiplicative error term (mean of 0 and a variance noted σ1) and the additive error term (mean of 0 and a variance noted σ2), respectively.

#### Pharmacokinetic Modeling

The pharmacokinetic data were analyzed in accordance with Guideline on reporting the results of population pharmacokinetic analyses (12). The theoretical blood sampling time points related to the preceding administration were used except when the difference between the actual time of collection and the planned time of collection exceeded +/−5%. At time points in the lag-time between time zero and the first drug concentration equal or above Limit of Quantification (LOQ), drug concentrations below LOQ were set to zero. Trailing concentrations below LOQ were not used in calculations.

The simplest model tested was composed of one central compartment with an absorption compartment. This model was then completed with additional peripheral compartments until the addition of a new compartment failed to significantly improve the model. Bioavailability was estimated precisely in a previous study (5) and used as an input in the current model (value fixed to 0.98).

#### Pharmacodynamic Modeling

According to the mechanism of action, the PD modeling was performed using the quantity of torasemide in urine (Q_urine_: quantity of torasemide excreted in urine in μg per day), rather that torasemide plasma concentrations since the sites of action of torasemide are located in the luminal side of the loop of Henle.

##### PK/PD modeling of diuresis

Daily production of urine volume was used for the quantitative analysis of the effect of torasemide on diuresis. The natural diuresis was taken into account in the model with a baseline value. The effect of torasemide on diuresis was modeled with a direct response model. The effect model tested were a sigmoid-E_max_ model, an E_max_ model, a power model and a linear model.

Diuresis model using a power model with a baseline effect parameter was modeled as follows (Equation 1).

(1) $$\begin{array}{r} E_{Diuresis}=Baseline+{slope\;*\;Q_{urine}}^{\alpha_{Tora}} \end{array}$$

Where, *E*_*Diuresis*_ is the measured effect (i.e., urine volume, in mL/day). Baseline is the urine volume produced over 24 h without treatment. α_*Tora*_ is the power parameter (unitless) and *slope* is the proportional factor of the effect of torasemide on diuresis (log_10_ mL/μg). The unit in log10 mL/μg, so a value of −4.11 corresponds to 10^−4.11^ mL/μg or 0.0000776 mL/μg. The model performance was assessed by the objective function value, the goodness of fits plots, the plausibility of parameter estimates and their precision.

##### PK/PD modeling of natriuresis

The effect of torasemide on natriuresis could not be modeled with direct response model due to the shape of the natriuresis profile and to the existence of a negative feedback (see below). Therefore, the natriuresis was modeled with an indirect response model. The quantity of sodium (Q_Na_, expressed in mEq) excreted was modeled as follows (Equation 2).

(2) $$\begin{array}{r} \frac{dQ_{Na}}{dt}=E_{Na} \end{array}$$

Where E_Na_ is the formation rate of sodium in urine (natriuresis, expressed in mEq/h). The bladder was considered empty after each urination (i.e., urine collection).

Without drug effect, the rate of change of natriuresis (in mEq/h^2^) was modeled with the following equation (Equation 3).

(3) $$\begin{array}{r} \frac{dE_{Na}}{dt}=K_{forNa}-\;k_{absNa}\;*\;E_{Na} \end{array}$$

Where, k_forNa_ in is the zero-order rate constant (mEq/h^2^) that increases natriuresis and k_absNa_ is the first-order rate constant (expressed in h^−1^) that decreases natriuresis.

The baseline natriuresis at steady state in the absence of drug effect is expressed as in Equation 4:

(4) $$\begin{array}{r} E_{Na}\left(steady\;state\right)=Baseline=\frac{k_{forNa}}{k_{absNa}} \end{array}$$

The effect of torasemide on natriuresis was fitted with four rival models: linear, power, simple E_max_ and Sigmoid-E_max_ model. As an example, Equation 5 represents the Sigmoid-E_max_ model:

(5) $$\begin{array}{r} E_{NaTora}=\;\frac{E_{maxNa}\;*\;{Q_{urine}}^{\alpha_{Tora}Na}}{{EC_{50\text{\_}apparent}}^{\alpha_{Tora}Na}+\;{Q_{urine}}^{\alpha_{Tora}Na}} \end{array}$$

Where, E_maxNa_ is the maximum drug effect on natriuresis (proportional increase, expressed in unitless dimension) and EC_50_apparent_ is the daily quantity of torasemide excreted in urine (expressed in μg, as Q_urine_) that produces 50% of the maximal effect and α_*Tora*_*Na* is the Hill coefficient (slope, unitless).

Torasemide is known to increase the natriuresis therefore, the effect was modeled as an increase of the k_forNa_ parameter and the equation of natriuresis became (Equation 6):

(6) $$\begin{array}{r} \frac{dE_{Na}}{dt}=k_{forNa}\;*\;\left(1+E_{NaTora}\right)-\;k_{absNa}\;*\;E_{Na} \end{array}$$

A reversible resistance (proportional decrease bounded between 0 and 100%, unitless dimension) of the diuretic effect also called “diuretic resistance” is described in the literature (13). It corresponds to a negative feedback that limits sodium excretion in urine. This resistance mechanism was included into the model (Figure 1), with an indirect response model (Equation 7).

(7) $$\begin{array}{r} \frac{dR_{Na}}{dt}=k_{ResON}-\;k_{ResOFF}\;*\;R_{Na} \end{array}$$

Where, k_ResON_ is the zero-order rate constant (h^−1^) for activation of the resistance and k_ResOFF_ is the first-order rate constant (h^−1^) for the loss of resistance. No resistance seemed to be active for low doses of diuretics and the model could not be fitted without a resistance threshold. Consequently, the resistance was considered activated when the effect of torasemide on rate of sodium excretion E_NaTora_ (untitless) was above a threshold value, estimated as a parameter of the model, otherwise k_resON_ was assigned a value of 0 (only at the lowest dose).

The resistance to diuretics decreases the effect of the drug on natriuresis and limits the sodium excretion. The effect of the resistance was modeled as a decrease in k_forNa_ or an increase in K_absNa._ Since k_forNa_ was dependent on the torasemide effect (E_NaTora_), the diuretic resistance could be modeled as a modulation in E_NaTora_ (Equation 4) through either an increase in EC_50_ (lower potency) or as a decrease in E_max_ (lower efficacy) as described in Equation 8:

(8) $$\begin{array}{r} E_{MaxNa\text{\_}apparent}=E_{Max\text{\_}baseline}\;*\;\left(1-{R_{Na}}^{\;}\right) \end{array}$$

Where, E_Max_baseline_ is the value of E_Max_ without resistance.

### Simulation of Dose-Effect Relationship

The simulation was performed with Berkeley Madonna® version 8.3 (University of California, USA). Simulations of diuresis effect were conducted at different doses of torasemide. The simulations were performed using the population PK/PD model developed in this paper, using the typical parameter to simulate the average effect obtained per dose.

## Results

### Pharmacokinetic Model

**Figure 2:**
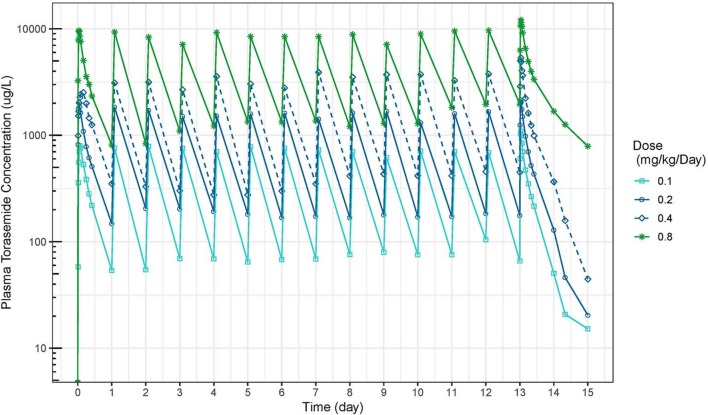
Torasemide plasma concentration-time (μg/L) curve after daily administration for 14 days at doses of 0.1, 0.2, 0.4, and 0.8 mg/kg (Study 1).

**Figure 3:**
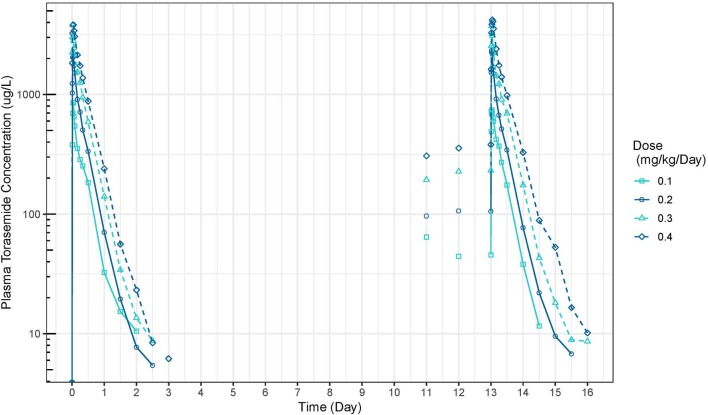
Torasemide plasma concentration-time curve (μg/L) after daily administration on day 1 and daily5 and 14 at doses of 0.1, 0.2, 0.3, and 0.4 mg/kg (Study 2).

**Figure 4:**
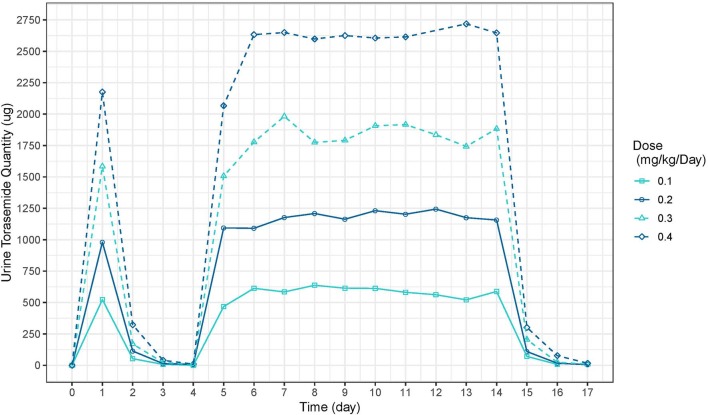
Torasemide urine quantity-time curve (μg) after daily administration on day 1 and daily between days 2, 5 and 14 at doses of 0.1, 0.2, 0.3, and 0.4 mg/kg (Study 2).

The mean plasma concentration-time and urinary excretion-time profiles of torasemide following oral administration once a day at 0.1, 0.2, 0.4, and 0.8 mg/kg/day in dogs (Study 1) are presented in Figure 2 (log scale) and in Figure S14, respectively. The mean plasma concentration-time and urinary excretion-time profiles of torasemide following single oral administration of 0.1, 0.2, 0.3, and 0.4 mg/kg/day on day 1 and subsequently between day 5 to 14 (Study 2) are presented in Figure 3 (log scale) and Figure 4, respectively.

A model with 2 compartments was chosen to describe the pharmacokinetics of torasemide over a compartmental model (OFV: −142) (Figure 1). The schematic illustration of the final structural model is presented in 1. The estimated population pharmacokinetic parameters of torasemide obtained with the final model are presented in Table 2. The goodness of fits plots, used to assess the final model performance, are presented in Figures S1–S3 for plasma and Figures S4–S6 for urine. Body clearance, when scaled by an average body weight of 10 kg, was 7.7 mL/kg/h, with a urinary clearance of 4.7 mL/kg/h, corresponding to low clearances as per (14). Urinary clearance (Fu) was estimated as 61% of total clearance and model analysis was not able to find a difference between doses for this parameter.

**Table 2:** Torasemide population pharmacokinetics parameters.

**Parameter**	**Unit**	**Description**	**Typical value (RSE%)**	**Inter-Individual variability CV% (RSE%) [*Shrinkage*]**
F		Bioavailability	0.98 FIX*	–
k_a_	/h	Absorption constant rate	1.66 (18.7%)	126.1% (11.5%) [*49.9%*]
CL	L/h	Total Clearance	0.077 (11%)	64.3% (16.6%) [*47.3%*]
Fu		Urine fraction of the total clearance	0.611 (2.4%)	8.6% (14.7%) [*10.1%*]
Vd	L	Volume of distribution of the central compartment	0.145 (23.9%)	–
Q	L/h	Clearance of transfer between compartments	0.262 (16.8%)	–
VdPER	L	Volume of distribution of the peripheral compartment	0.935 (4.6%)	–
Proportional error residual in plasma (%)			18.4% (13.8%)	–
Proportional error residual in urine (%)			22.5% (12.1%)	–
Additive error residual in urine (μg)			3.68 μg (41%)	–

* *Estimated precisely in a previous study. CV, Coefficient of variation; RSE, Relative Standard Error*.

*F, bioavailability (fixed from unpublished study reported in European Public Assessment Report (5), k_a_, constant rate of absorption; Vd, volume of distribution of the central compartment; Q, clearance between central and peripheral compartment; VdPER, volume of distribution of the peripheral compartment; CL, the total body clearance and fu, urine fraction of the total clearance*.

For all doses, the accumulation ratio was not different from unity. However, torasemide plasma AUC did not statistically verify dose proportionality when assessed with a power model $\left(Y=\;\alpha \;\times \;{AUC_{0-\infty }}^{\beta }\right)$, β was 1.26 for the single dose (90% Confidence interval 1.17–1.35) and 1.59 (90% CI 1.30–1.88) for the repeated dose.

### Pharmacodynamic Effects: Diuresis

#### Torasemide and Furosemide Diuretic Profiles

**Figure 5:**
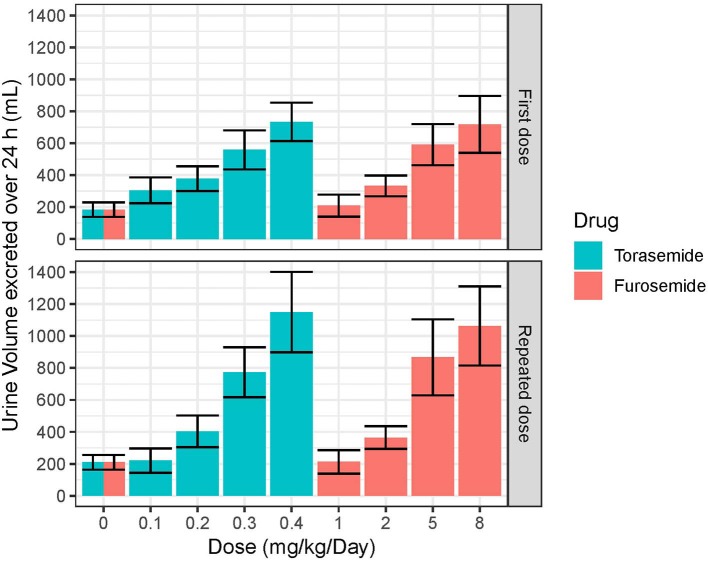
Mean and SD urine volumes (mL) excreted over 24 h obtained after single or daily repeated oral administrations of 0.1, 0.2, 0.3, or 0.4 mg/kg/day of torasemide once a day or 1, 2, 5, or 8 mg/kg/day of furosemide (in 2 daily administrations) in dogs of study 2.

**Figure 6:**
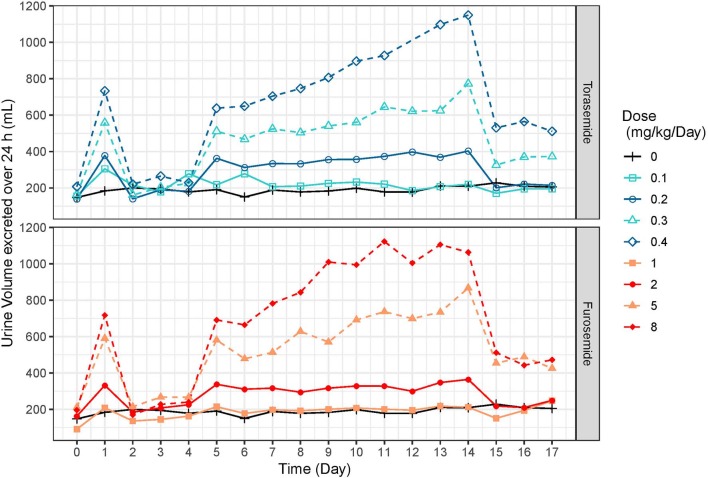
Mean urine volumes (mL) excreted over 24 h profiles obtained after daily repeated administrations of 0.1, 0.2, 0.3, or 0.4 mg/kg/day of torasemide or 1, 2, 5, or 8 mg/kg/day of furosemide (in 2 daily administrations) in dogs of study 2.

The mean and SD urine volumes excreted over 24 h obtained after first or repeated oral administrations of torasemide (0.1 to 0.4 mg/kg/day, in one daily dose) or furosemide (1 to 8 mg/kg/day, in 2 daily doses) in dogs from study 2 are presented in Figure 5 (first vs. repeated dose diuresis) and Figure 6 (daily diuresis for 14 days).

Following oral administrations of torasemide and furosemide, urine volume excreted over 24 h increased with doses. This relationship urine volume vs. dose was observed either after a single dose or after repeated doses.

The average urine volume excreted per day was relatively constant over time, with repeated doses of torasemide up to 0.2 mg/kg/day and with repeated doses of furosemide up to 2 mg/kg/day but kept increasing for higher doses of either drugs.

With repeated 0.3 mg/kg/day doses of torasemide, the average volume increased from 512 mL after the first administration to 625 mL after the 9^th^ dose and 773 mL after the 10^th^ dose. With repeated 0.4 mg/kg/day doses of torasemide, the average urine volume increased after the 3^rd^ dose from 638 mL after the first administration to 1,150 mL after the 10^th^ dose. With repeated doses of furosemide at 5 mg/kg/day, the average urine volume increased from 581 mL after the first administration to 867 mL after the 10^th^ day of administration. With repeated dose of furosemide at 8 mg/kg/day, the average urine volume increased after the 3^rd^ day onwards, from 691 mL after the first administration to 1,063 mL after the 10^th^ dose.

A low to moderate intra-individual variability of the diuretic effect was observed up to 0.2 mg/kg/day torasemide (CV at 26.9 and 29.4% for 0.1 and 0.2 mg/kg/day, respectively) and up to 2 mg/kg/day furosemide (CV at 23.9 and 24.1% for 1 and 2 mg/kg/day, respectively). Diuresis increases at higher doses of torasemide (0.3 and 0.4 mg/kg/day, with CV at 37.9 and 43.5%, respectively) and furosemide (5 and 8 mg/kg/day, with CV at 38.3 and 45.5%, respectively) were highly variable among dogs.

At the end of the treatment period, the urine volume decreased rapidly and returned to baseline in one day for doses up to 0.2 mg/kg/day torasemide and 2 mg/kg furosemide. For higher doses, the daily urine volume decreased at the end of the treatment to a steady state higher than the original baseline (220 mL/day). This was ~370 mL at 0.3 mg/kg/day and 510 mL at 0.4 mg/kg/day for torasemide and ~450 mL for both high doses furosemide. The measurements were only continued for 2 days beyond cessation of dosing, so the kinetic of resumption of normal diuresis at these doses is unknown. However, the study was conducted with a cross-over design and the baselines measured at the beginning of each period were similar, indicating that these higher diuresis levels returned to the original baseline before the beginning of the next treatment period (washout 2 weeks).

#### PK/PD Modeling of Torasemide-Induced Diuresis

A power model was chosen to describe the pharmacodynamic diuretic effect of torasemide over the linear (OFV: −83), simple E_max_ (OFV: −83), and sigmoid E_max_ (OFV: −1,202) models (Figure 1). The estimated population pharmacodynamics parameters of torasemide on diuresis obtained with the final PD model are presented in Table 3. The goodness of fits plots, used to assess the final model performance, are presented in Figures S7–S9.

**Table 3:** Population pharmacodynamics parameters of torasemide-induced diuresis.

**Parameter**	**Units**	**Description**	**Typical Value (RSE%)**	**Inter-Individual variability CV% (RSE%) [*Shinkage*]**
Baseline	mL/day	Volume of urine produced over 24 h without treatment	221 (4.0%)	13.4% (18%) [*11%*]
slope	log_10_ mL/μg	Proportional factor of the effect of Torasemide on diuresis	−4.11 (13%)	45.8% (15%) [*26%*]
α_*Tora*_	–	Power parameter	2.05 (9%)	3.9% (36%) [*49%*]
Proportional error residual (%)			43.2% (4%)	

*Where, E_Diuresis_ is the measured effect (i.e., urine volume, in mL/day)*.

$$\begin{array}{l} E_{Diuresis}=Baseline+\;{slope\;*\;Q_{urine}}^{\alpha_{Tora}} \end{array}$$

*Baseline is the urine volume produced over 24 h without treatment. α_Tora_ is the power parameter (unitless) and slope is the proportional factor of the effect of torasemide on diuresis (mL/μg). The unit in log_10_ mL/μg, so a value of −4.11 corresponds to 10^−4.11^ mL/μg or 0.0000776 mL/μg. The model performance was assessed by the objective function value, the goodness of fits plots, the plausibility of parameter estimates and their precision*.

### Pharmacodynamic Effects: Natriuresis

#### Torasemide and Furosemide Natriuretic Profiles

**Figure 7:**
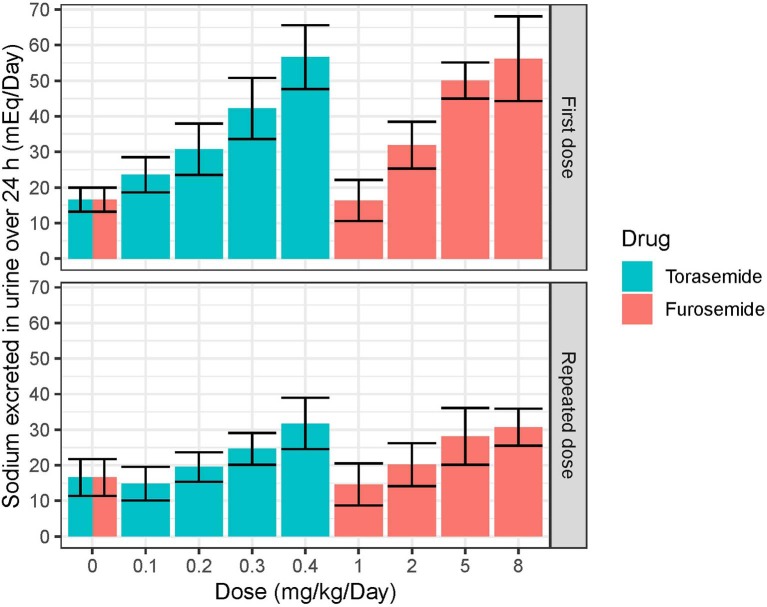
Mean and SD quantities of sodium (mEq/day) excreted over 24 h obtained after single or daily repeated oral administrations of 0.1, 0.2, 0.3, or 0.4 mg/kg/day of torasemide once a day or 1, 2, 5, or 8 mg/kg/day of furosemide (in 2 daily administrations) in dogs of study 2.

**Figure 8:**
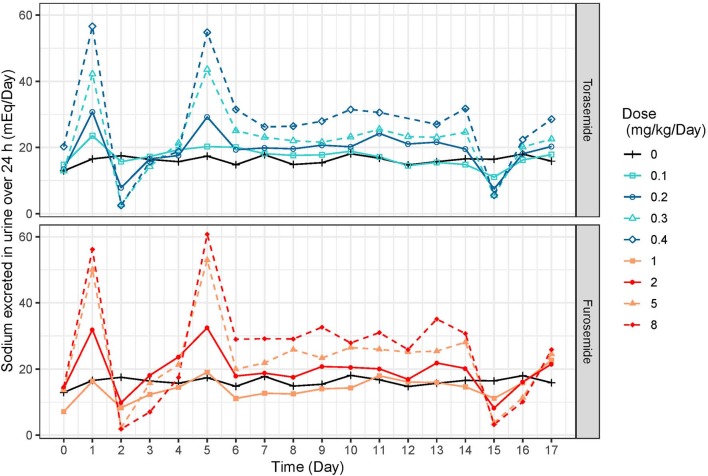
Mean daily natriuresis time-profiles (mEq/day) profiles obtained after daily repeated administrations of 0.1, 0.2, 0.3, or 0.4 mg/kg/day of torasemide or 1, 2, 5, or 8 mg/kg/day of furosemide (in 2 daily administrations) in dogs of study 2.

Following first oral administration of torasemide and furosemide, the quantities of sodium excreted over 24 h increased with dose (Figure 7). From a baseline of 16 mEq/day, torasemide and furosemide rose natriuresis to 56.6 and 56.2 mEq/day, with their respective maximal doses. Natriuresis decreased dramatically after the 2^nd^ and 3^rd^ treatment days of study 2 and then remained stable until the end of the treatment period (Figure 8). For doses up to 0.3 mg/kg/day of torasemide, natriuresis decreased to a value near the baseline and for a 0.4 mg/kg/day dose, natriuresis decreased to a higher value (27 mEq/day) within 2 days after cessation of treatment. Similarly, with low doses of furosemide (up to 2 mg/kg/day), natriuresis decreased to values near the initial baseline values and for 8 mg/kg/day dose, natriuresis decreased to a value higher than baseline (28 mEq/day). At the end of the treatment period, a temporary decrease in the natriuresis below the baseline (sodium retention) was observed in all dogs and for all doses with both drugs.

#### PK/PD Modeling of Torasemide-Induced Natriuresis

The sigmoid E_max_ model outperformed the linear (OFV: −73), power (OFV: −72) and simple E_max_ (OFV: −119) models (Figure 1). This indirect response model related the quantity of torasemide excreted in urine to the effect to a stimulation of sodium loss (K_forNa_). The maximal effect (E_max_) was 4.67 and the quantity of torasemide excreted in urine that produces 50% of the maximal effect EC_50_ was 1,080 μg of urinary torasemide per day.

A “diuretic resistance” mechanism was modeled with an indirect response model where torasemide urinary excretion fractionally decreased the maximal effect E_max_ when E_NaTora_ exceeded a fractional threshold of 0.055 or 5.5%. This means that when the rate formation of natriuresis E_NaTora_, exceeds 5.5% of the baseline value, the resistance will be triggered. Practically, only the lowest dose did not reach threshold. For modeling diuretic resistance in natriuresis, modeling loss of efficacy (fractional reduction of E_max_) outperformed decrease in potency (fractional increase in EC_50_, OFV −100) or increase in effect dissipation (increase in k_absNA_, OFV −58,609).

The estimated population pharmacodynamics parameters of torasemide on natriuresis obtained with the final PD model are presented in Table 4. The goodness of fit plots, used to assess the final model performance, are presented in Figures S10–S12.

**Table 4:** Population pharmacodynamics parameters of torasemide-induced natriuresis.

**Parameter**	**Units**	**Description**	Typical Value (RSE%)*	**Inter-Individual variability CV% (RSE%) *[Shinkage]***
k_forNa_	mEq/h^2^	Zero-order rate constant for production of natriuresis	3.67 (3.65%)	19.4% (28.30%) *[29%]*
k_absNa_	/h	First-order rate constant that decrease the formation of natriuresis	4.99 (5.76%)	–
E_maxNa_	Unitless	Maximum drug effect on natriuresis	4.67 (10.53%)	62.4% (32.44%) *[1%]*
EC_50Na_	μg	Quantity of torasemide excreted in urine that produces 50% of the maximal effect	1080 (5.97%)	–
α_*Tora*_*Na*	–	Factor of sigmoidicity	2.57 (12.50%)	–
k_resOn_	/h	Zero-order rate constant for activation of the resistance	0.0811 (12.94%)	–
k_resOff_	/h	First-order rate constant for the loss of resistance	0.0894 (11.21%)	-
Threshold	Unitless	Threshold of activation of resistance	0.0547 (12.31%)	–
Proportional error residual (%)			30.5% (7.69%)	–

*E_Na_ is the formation rate of sodium in urine (natriuresis, expressed in mEq/h), k_forNa_ is the zero-order rate constant (mEq/h^2^) that increase the natriuresis and k_absNa_ is the first-order rate constant (expressed in h^−1^) that decrease the natriuresis. The effect of torasemide on natriuresis best fitted a sigmoid-E_max_ model, where, E_maxNa_ is the maximum drug effect on natriuresis (unitless) and EC_50_apparent_ is the daily quantity of torasemide excreted in urine (expressed in μg, as Q_urine_) that produces 50% of the maximal effect and α_Tora_Na is the Hill coefficient (slope, unitless). Torasemide is known to increase the natriuresis therefore, the effect was modeled as a fractional increase of the k_forNa_ parameter. k_ResON_ is the zero-order rate constant (h^−1^) for activation of the resistance and k_ResOFF_ is the first-order rate constant (h^−1^) for the loss of resistance. The resistance was active when the effect of torasemide on rate of sodium excretion E_NaTora_ (untitless) was above a threshold parameter estimated by the model (otherwise k_resON_ = 0). The effect of the resistance was modeled as a decrease in k_forNa_. Since k_forNa_ was dependent of the torasemide effect (E_NaTora_), the diuretic resistance could best be modeled as a modulation in E_NaTora_ (Equation 4) through a decrease in E_max_ (lower efficacy)*.

* *RSE: Relative Standard Error*.

### Aldosterone

**Figure 9:**
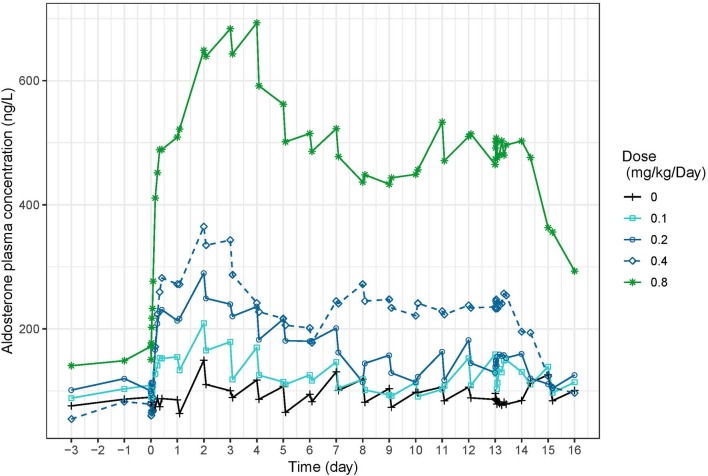
Mean serum aldosterone concentration (ng/L) time-profiles profiles obtained before and 2 h after daily repeated administrations of 0.1, 0.2, 0.3, or 0.4 mg/kg/day of torasemide in dogs of study 1.

Aldosterone concentrations increased with dose (Figure 9). In study 1 (Figure 1), peak aldosterone concentrations were reached at 48 h and concentrations progressively came back to baseline with the 0.1 and 0.2 mg/kg dose, whereas they stabilized around 250 ng/L and 450–500 ng/L from day 7 to 14 for doses of 0.4 and 0.8 mg/kg, respectively. After a single day administration (study 2, Figure S13), the mean serum concentration of aldosterone increased dose-proportionally up to 24 after dosing with all the doses of torasemide and the two higher doses of furosemide. The peak plasma aldosterone observed at 24 h ranged from 27.3 to 141.1 ng/L for doses of 0.1 and 0.4 mg/kg/day of torasemide and was 109.5 and 155.1 ng/L for the two higher doses of furosemide (5 and 8 mg/kg/day). The serum concentrations then decreased and reached the baseline level 96 h after single day administration with each of the doses. At steady state, just before the last administration day (day 14), serum aldosterone concentrations were practically at baseline levels with the two lowest doses of torasemide and furosemide. The concentrations of aldosterone were 69 and 120 ng/L with 0.3 and 0.4 mg/kg/day doses of torasemide and 222 to 261 ng/mL with 5 and 8 mg/kg/day of furosemide.

### Dose Determination

**Figure 10:**
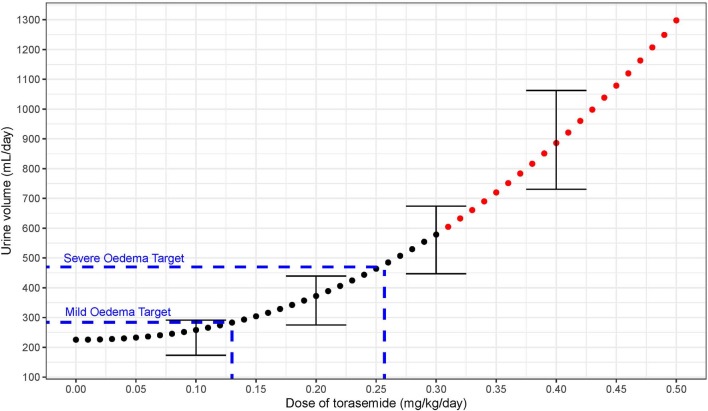
Dose response relationship between diuresis (mL/day period) and doses of torasemide in dogs (mg/kg/day) as computer from the PK/PD model. The variability observed with real data is represented with error bars (Doses with high variability in effect plotted in red).

First, an abacus (Table 5) was designed from the observed data at day 9 to understand the dose correspondence between furosemide and torasemide for diuresis effect. The Torasemide/Furosemide dose ratio for equivalent daily diuresis was approximately 1/10 at doses up to 0.2 mg/kg of torasemide and reached 1/20 at the highest doses. The torasemide dose-response relationship for diuresis (24 h period) in dogs was simulated from the PK/PD model and is presented in Figure 10. Higher inter-individual variability was observed from doses equal or higher than 0.3 mg/kg/day.

**Table 5:** Corresponding doses of furosemide and torasemide for diuresis effect.

Furosemide Dose (mg/kg/day)	0	1	2	5	8
Diuresis obtained at D9 (mL/day)	210	212	365	867	1,063
Torasemide Dose (mg/kg/day)	0	0.1	0.2	0.3	0.4
Diuresis obtained at D9 (mL/day)	210	220	403	773	1,150

## Discussion

### PK and PD of Torasemide

Torasemide was rapidly absorbed after oral administration; with peak plasma concentrations obtained within 1 h following administration. Absorption half-life (25 min) was identical to the one reported by Paulin et al. (15). The bioavailability value (98% estimated from a previous unpublished study, reported in the European Public Assessment Report (5) inputted in the model was in line with the one estimated by Paulin et al. (91.8%). Torasemide clearance estimate (7.7 mL/kg/h) was slightly lower than Paulin et al. (12.4 mL/kg/h). In healthy Beagles with normal renal function, our estimate of 61% of the dose of torasemide eliminated unchanged in the urine differed slightly from the one from Paulin et al. (80%) (15). Torasemide's longer elimination half-life (around 6 h vs. 3 h for furosemide, measured in the present study by results not shown), combined with higher potency and extensive urinary excretion makes it appropriate for dosage every 24 h. As a consequence, diuresis induced given a once a day administration of torasemide was the same as with furosemide given at an equivalent dosage in 2 administrations. Moreover, no accumulation occurred after 14 days, even though PK deviates from linearity at higher doses.

Using the quantity of torasemide in urine as factor supporting the activity provided a much better fit than modeling with plasma concentrations and was the most physiologically relevant option as torasemide is secreted into the proximal convoluted tubule through organic anion transporters (4). Contrary to Paulin et al. (15), who modeled torasemide PK/PD relationship over 96 h following a single administration and used a 5 day diuresis dataset from another set of dogs to validate the model, the present model pools intensely sampled PK and PD information from 17 dogs treated for 14 days with a range of doses bracketed within clinically relevant options. Longer treatment and more intense monitoring increase our confidence in the immediate effects and body adaptation to torasemide treatment.

Torasemide-inducted diuresis was comparable to a previous study (16), where dogs were administered 0.2 mg/kg torasemide twice a day (60 and 90 mL/kg/day after day 1 and 14, respectively). In the present study, diuresis after 10 treatment days were higher than after a single treatment day for high doses torasemide (≥0.3 mg/kg/day), whereas for doses up to 0.2 mg/kg/day, the daily diuresis remained constant over time. Similar time-dependent increase was observed with doses of furosemide ≥5 mg/kg/day. Since the quantity of torasemide excreted in urine was constant over time, no accumulation of torasemide could explain this increase. The increase of diuresis over time at doses ≥0.3 mg/kg/day was highly variable between dogs; therefore we recommend to limit the administrations at doses higher than 0.3 mg/kg/day for a 3–5 days maximum period to prevent an excessive diuretic effect in some dogs that respond highly to the drug. Of note, the same increase in variability was observed with furosemide at doses above 5 mg/kg. While additional experiments are warranted to ascertain the clinical relevance of this finding on the optimal furosemide dosing regimen, this may be kept in mind while using furosemide on clinical cases.

Natriuresis increased after the first dose after starting repeated torasemide administration, then decreased dramatically after the 2^nd^ and 3^rd^ treatment days and remained constant until the end of the treatment period. For doses up to 0.3 mg/kg/day, natriuresis decreased to a value close to the baseline value and for 0.4 mg/kg/day dose, natriuresis decreased to a slightly higher value than the baseline one (27 mEq/mL). Similar profiles were also observed with furosemide. The decrease in natriuresis could only be modeled with inclusion of a reversible threshold-activated resistance and was not associated with changes in diuresis, leading to typically observed increasingly hypotonic urine production (4). Progressive loss of natriuresis is due to a combination of various possible mechanisms resulting in what is called “diuretic resistance.”

### Resistance to Natriuresis Induced by Loop-Diuretics

During each treatment period, the sodium excretion was the result of 3 mechanisms: i) the basal excretion of sodium (sodium intake minus renal and non-renal losses), ii) the torasemide effect and iii) the “diuretic resistance” for which the natriuretic component was empirically captured by the model. At the end of each treatment period, the torasemide urine concentration decreased quickly, leading to a rapid offset of its natriuretic effect. Due to the slower reversion of natriuretic resistance, sodium urinary excretion briefly fell below the original baseline the day after the last dose. The magnitude of the negative overshoot was commensurate with the dose-dependent intensity of natriuretic resistance for both drugs. The sodium excretion returned to its original baseline after a limited amount of time (around 24 h) confirming the rapid reversibility of the mechanism of natriuretic resistance induced by loop diuretics.

Real or apparent loop diuretic resistance can occur because of 3 possible mechanisms in hypervolaemic human patients (17). The most common is what is understood as rebound sodium retention, where blockade of sodium reabsorption at the loop of Henle leads to hypertrophy of the distal sites of the nephron and pronounced sodium reabsorption through increased expression of Na^+^/Cl^−^ cotransporter located in the distal convoluted tubule (18). A second mechanism is a post-diuretic effect aiming at protection of the intravascular volume, is likely mediated by activation of angiotensin II or sympathetic nervous system (17). This compensatory sodium-retention effect is exacerbated when the diuretic action wanes before the next dose and urinary concentrations are low. More sustained exposure to loop diuretics may prevent the second mechanism and have been explored in large scales randomized trials as the DOSE trial (19) which compared continuous infusion and intermittent boluses in 308 patients in acute decompensated heart failure (see infra). The last mechanism, which may occur concomitantly with the two previous ones, is “diuretic braking” or tolerance. It is a true loss of patient's response to diuretic after the first dose, depending on the degree of volume and sodium depletion.

Jardim et al. (20) describe in details the pathophysiology of diuretic resistance, in particular the neuro-hormonal activation. Hyponatraemia and activation of the Renin Angiotensin Aldosterone System (RAAS) could have led to the observed dose-dependent aldosterone response. RAAS could have been activated in a volume independent pathway in the MD because of the loss of the link between tubular chloride and inhibition of renin secretion. The sensing is through chloride co-transport which is blocked by torasemide/furosemide, hence activation of renin secretion occurs very quickly following administration of either drug. The other possible activation pathway is through volume contraction (vascular stimulus) (13, 21). RAAS activation leads to sodium retention through promotion of tubular compensatory adaptation, post-diuretic sodium retention and diuretic braking phenomenon.

### What Dose Is Necessary for Efficient Decongestion in Acute Congestive Heart Failure?

Congestion is defined as the signs and symptoms of extracellular fluid accumulation, instigated by an increase in left-sided cardiac pressures (22). The question of *a-priori* dose finding for a diuretic to achieve decongestion is a rare exercise and can be approached by i) targeting sufficient reduction of circulating blood volume or ii) matching net fluid losses empirically required to achieve euvolaemia.

Blood volume may be increased by as much as 30% in dogs with severe heart failure (23) and it could be tempting to consider primary reduction in blood volume to inform diuretic doses. However, one needs to distinguish between changes in total blood or interstitial tissue volume (volume overload) and changes in venous capacitance (volume misdistribution); their contribution to congestion may differ widely between patients (22). Miller and Mullan (24) measured total blood volume and plasma volume (radiolabeled-albumin dilution) in 26 patients with decompensated chronic heart disease before and after diuretic therapy. There was substantial variability in the source and quantity of fluid removed with respect to intravascular and interstitial compartments. Compared to expected normal blood volume (5.3 +/– 0.7 L), total blood volume was 39% increased on admission (7.4 +/– 1.6 L, range 9.5 to 107% increase) and still 30% increased on discharge (6.7 +/- 1.3 L, range 9–51% increase). The major component of the volume loss (85%) was derived from the interstitial space (calculated as 6.2 +/– 0.4 L) and not the plasma (15%) and definitely precludes diuretic dose determination based on circulating volume reduction.

On the other hand, there is a clear inverse relationship between the immediate efficacy of decongestion (urine output and net fluid loss) and short-term survival. In a sample of 475 patients with acute decompensated heart failure (ADHF), both net fluid loss (sum of daily fluid intake minus total output) and urine output following furosemide treatment were dose related amongst other variables and correlated with 6 months mortality (25). Mean urine output was 2.7 L after 24 h with a net fluid loss was 1 L per 24 h, corresponding to 14.2 mL/kg/day for an average 70 kg human patient. Response to diuretic in patients with ADHF involves a complex and dynamic process; dose was the strongest predictor of urine output, but only explained 50% of the variability in diuresis, the rest of the variability being explained by renal function, hemodynamic statues, degree of initial volume overload and fluid intake.

Despite ubiquitous use of loop diuretics in human patients, there is only one randomized controlled trial (Diuretic Optimization Strategies Evaluation DOSE trial) that has prospectively compared the effect of dosage regimen in the treatment of acute heart failure (19). A sample of 308 patients were randomly assigned to one treatment of the 2 × 2 factorial design including dose (low dose 1 × vs. high dose 2.5x) and dose intensity (repeated bolus vs. continuous infusion). Higher doses were associated with higher diuresis and improved relief from dyspnea and were not associated with worse clinical outcome at 60 days. The mean 72 h-cumulated net fluid loss were 3.6 L and 4.9 L, which correspond to average daily net fluid losses (70 kg patient) of 17 and 23 mL/kg/day for the low and high doses, respectively. Extrapolating to a 10.5 kg dog with a model-estimated baseline diuresis of 220 mL/day (Table 5), low and high dose decongestion target additional urine volumes of 180 mL (target daily diuresis = 180 + 220 = 400 mL) and 240 mL (target daily diuresis = 460 mL) for the low and high dose decongestion targets, respectively. Another study (CARRESS-HF) compared outcomes between conventional diuretic therapy and mechanical ultrafiltration (which involves removal of iso-osmotic filtrate) in 188 patients with ADHF (26). Aggressive diuretic titration followed a urine output-guided protocol, targeting 3 to 5 L per day. Cumulative fluid loss were averaging 3 L for the first 24 and remained constant until 96 h where cumulated fluid loss was 12 L. Assuming baseline daily diuresis of 1.4 L for a normal 70 kg person, daily additional urinary losses during the trial were a 22.8 mL/kg/day, yielding by extrapolation an additional 240 mL diuresis for a 10.5 kg dog.

Extrapolating from the DOSE (19) and CARRESS-HF (26) studies, the minimal dose required to reduce the congestion in the dog would eliminate 230 mL of urine in addition to the normal diuresis (i.e., 240 mL + 220 mL = 460 mL). According to our PK/PD modeling approach, the minimal recommended dose to treat moderate to severe pulmonary oedema associated with congestive heart failure due to degenerative mitral valve disease in dog would be 0.26 mg/kg/day (3.5 mg/kg/day furosemide equivalent),. One limitation is that the studies were carried out in healthy dogs. Torasemide oral bioavailabiltiy may not be that high in human patients and dogs due to gastrointestinal oedema, gastroparesis, and delayed gastric emptying (17), or the possibly lowered of distribution of diuretic to the nephron lumen with a cardiorenal syndrome type 1 (decompensated heart failure leading to renal hypoperfusion).

### Dose for Milder Congestion and Maintenance Dose After Decongestion

Excess of fluid volume from canine experimental models of mild pulmonary oedema induced chemically with either oleic acid (27) or acetylene (28) was estimated to an average of 64 mL. According to the PK/PD model (Figure 10), the dose of torasemide that corresponds to the sum of baseline diuresis and excess water in these mild induced oedemas (220 + 64 = 284 mL/day) would be 0.13 mg/kg/day. One persistent problem with treating human patients and dogs who recover from congestion with volume overload, is knowing when euvolaemia is reached and diuretic dose should be reduced. The ideal maintenance dose to maintain sustained decongestion would be one that achieves excretion of isotonic urine with modest diuresis driven by torasemide urinary excretion that are below the threshold of activation of natriuresis resistance and neuro-hormonal activation. A dose of torasemide of 0.1 mg/kg/day does not activate an aldosterone response lasting more than 5 days, but achieves a diuretic and natriuretic effect only slightly above baseline in healthy dogs. A dose between 0.1 and 0.2 mg/kg/day (furosemide equivalent to 1–2 mg/kg/day) may be evaluated in a clinical field trial as an effective dose for milder congestion and chronic control of extracellular fluid volume.

One limitation of the study is that we were not successful in implementing a systems pharmacology approach, where natriuresis, diuresis, aldosterone, as well as variables which were measured in these studies but not reported in the present paper (kaliuresis and GFR) and PK would be included in the same model. It was however impossible due to the accumulation of small bias, like relying on spontaneous micturition. Urine was collected by physiologic urination, to avoid any additional stress or pain that could occur with catheterization. However, physiologic urination does not directly measure the urine formation by the kidney in the bladder but the urine formation between two consecutive urinations. Therefore, the urine data would be dependent of the interval of urination and could bias the results, especially when the urine collections are close (study 1). In order to avoid any bias, urine was analyzed over 24 h periods, so for each day, the urine volumes and the quantity of urinary sodium and torasemide were summed over 24 h periods.

The mechanism whereby urine volume, but not sodium excretion, increases at higher doses over the 14 day dosing period, leading to dissociation between sodium and water excretion is difficult to explain. A washout of the medullary interstitial sodium gradient could have led to inability to concentrate the urine. However, it is unclear (i) why water excretion continues to increase over time with either diuretics, (ii) what the therapeutic consequences are and (iii) what underlies the individual variability between dogs. The measurement of plasma ADH concentration, water balance and plasma osmolality over this dosing period would be interesting in future studies to understand this phenomenon better.

## Conclusion

Using the quantity of torasemide excreted in urine provided a much better prediction of torasemide pharmacodynamics than the plasma concentration-time course. A once-daily dosage regimen is supported by pooled PK/PD analysis of 2 preclinical studies with daily administration extending over 14 days. The dose ratio torasemide/furosemide decreased with dose (1/10 at doses ≤ 0.2 mg/kg/day torasemide to 1/20 at the highest dose of 0.4 mg/kg). For a daily target diuresis of 460 mL (decongestion of moderate to severe acute pulmonary oedema), a minimal dose of 0.26 mg/kg/day (3.5 mg/kg/day furosemide equivalent) is proposed for evaluation into clinical phases. Due to the high inter-individual variability in responses at doses ≥ 0.3 mg/kg, higher doses should be used for a limited period of time to prevent too high an excessive diuretic effect in some dogs that respond highly to the drug. Subsequently, a dose range of 0.1 to 0.2 mg/kg is proposed for mild pulmonary oedema or for long-term control of extracellular fluid volume. PK-PD modeling of preclinical data substantially de-risked dose finding; however the dose prediction capability of the model is directly linked to the reliability of the preclinical results and should be confirmed through pivotal clinical studies.

## Data Availability Statement

The datasets for this article are not publicly available because: they contain proprietary information of a newly registered veterinary drug. Requests to access the datasets should be directed to Mathieu Peyrou: mathieu.peyrou@ceva.com.

## Ethics Statement

The animal studies were reviewed and approved by ethical committees, and were performed in accordance to European Directive 2010/63/EU on the protection of animals used for scientific purpose.

## Author Contributions

JE: Preclinical expert for ISEMID® registration and contributed to the interpretation of results and manuscript content. EG: Contributed to data interpretation and manuscript content. AG: Responsible for the conduct of one the study (n°2). JG: Responsible for the conduct of one the study (n°1). RM: R&D Ceva Santé Animale, responsible for the bioanalytical analysis of both studies. MP: Contributed to data interpretation and manuscript content. LP: PK/PD expert and drafting of the manuscript. MJ: Responsible for the conduct of population PK analysis.

## Conflict of Interest

The authors declare that this study received funding from Ceva Santé Animale. The funder had the following involvement with the study: study design, collection, analysis and the decision to submit it for publication. These studies were conducted as registration studies for authorization of ISEMID®. JE and LP were paid consultants of the company.
