# Supplementary material for: Population Pharmacokinetics and Pharmacodynamics Modeling of Torasemide and Furosemide After Oral Repeated Administration in Healthy Dogs
Source: Front Vet Sci. 2020 Apr 28;7:151. doi: 10.3389/fvets.2020.00151 (PMC7199743; doi:10.3389/fvets.2020.00151)
Supplement: Supplementary file 1 [file Table_1.pdf]

Table S1: List of the PK/PD studies with subject demographics, study design and torasemide doses and blood / urine sampling schedule. In grey, day associated with dosing

Study	Subjects	Weight range (kg)	Age range (y.o.)	Number of periods	Doses	Design	Timing	Administration	Blood Sampling schedule	Urine sampling schedule
STUDY 1	5 male dogs	9.3 to 11.6 kg	1 to 2.1 yo	5 periods randomised crossover	**Torasemide** o r **Placebo**	Baseline		No treatment	**Day -3 and Day -1**: Once a day, in the morning	[0-24h] collections: from Day -3 to Day-2 and within 24h prior to drug administration
						Repeated daily doses	Day 1 (1st day of treatment)	Every day, Day 1 to 14	**Day 1**: T0h (before food intake) and then 15 min, 30 min, 45 min, 60 min, 90 min, 2 h, 4 h, 6 h, 8 h, 10 h after treatment	[0-24h] collection: after treatment during the following intervals:[0-2h], [2-4h], [4-6h], [6-8h], [8-10h], [10-12h], [12-24h].
							Day 2 -13		**Day 2 to Day 13**: T0h (before food intake) then 2 h post dosing	None
							Day 14 (last day of treatment)		**Day 14**: T0h (before food intake) and then 15 min, 30 min, 45 min, 60 min, 90 min, 2 h, 4 h, 6 h, 8 h, 10 h, 24 h, 32 h, 48 h, 52 h and 72 h after treatment administration.	[0-24h] collection: after treatment during the following intervals: [0-2h], [2-4h], [4-6h], [6-8h], [8-10h], [10-12h], [12-24h].

The daily treatmenst in STUDY 1 were torasemide (0.1, 0.2, 0.4 and 0.8 mg/kg, administered orally in the morning) or placebo. Washout was > 14 days

Study	Subjects	Weight range (kg)	Age range (y.o.)	Number of periods	Doses	Design	Timing	Administration	Blood Sampling schedule	Urine sampling schedule
STUDY 2	12 male dogs	9.9 to 11.2 kg	1.2 to 1.6 yo	9 periods randomised crossover	**Torasemide** or **Furosemide** or **Placebo**	Baseline		No treatment	**Day -3* and Day -1***: Once per day, in the morning (before feed intake).	[0-24h] collections: Day -3 and Day -1
						Single dose	Day 1 (1st day of treatment)	Day 1	**Day 1**: T0h (before feed intake) and then 15 min, 30 min, 45 min, 1h, 1.5h, 2h, 4h, 6h, 8h, 12h, 24*h after morning treatment.	Day 1: [0-12h], [12-24h] intervals after morning treatment (see Section 4 Deviations).
							Day 2 - 4	No treatment Day 2 to 4	**Day 2**: 36h, 48h* after Day 1 treatment<br>**Day 3**: 60h, 72h* after Day 1 treatment<br>**Day 4**: 96h* after Day 1 treatment	[0-24h] collection: from 24 to 48h after treatment<br>[0-24h] collection: from 48 to 72h after treatment<br>[0-24h] collection: from 72-96h after treatment
						Repeated daily doses	Day 5 - 13	Every day, Day 5 to 14	**Day 5 - 11**: No sample	[0-24h] collection: daily after morning treatment
									**Day 12 and 13**: Once per day, before morning treatment (before feed intake).	[0-24h] collection: daily after morning treatment
							Day 14 (last day of treatment)		**Day 14**: T0h (before feed intake) and then 15 min, 30 min, 45 min, 1h, 1.5h, 2h, 4h, 6h, 8h, 12h, 24h*, 36h, 48h*, 60h, 72h* and 96h* after morning treatment.	Day 14: [0-12h], [12-24h], [24-48h], [48-72h] and [72-96h] intervals last morning treatment

The daily treatments in STUDY 2 were torasemide (0.1, 0.2, 0.3 and 0.4 mg/kg, administered orally in the morning), furosemide (0.5, 1, 2.5 and 4 mg/kg/12h, administered orally in the morning and evening) or placebo

* Only serum on activated clotting tube
