# Supplementary material for: Population Pharmacokinetics and Pharmacodynamics Modeling of Torasemide and Furosemide After Oral Repeated Administration in Healthy Dogs
Source: Front Vet Sci. 2020 Apr 28;7:151. doi: 10.3389/fvets.2020.00151 (PMC7199743; doi:10.3389/fvets.2020.00151)
Supplement: Supplementary file 2 [file Table_2.pdf]

Supplementary Material

1 Supplementary Data

Supplementary Material should be uploaded separately on submission. Please include any supplementary data, figures and/or tables. All supplementary files are deposited to FigShare for permanent storage and receive a DOI.

Supplementary material is not typeset so please ensure that all information is clearly presented, the appropriate caption is included in the file and not in the manuscript, and that the style conforms to the rest of the article. To avoid discrepancies between the published article and the supplementary material, please do not add the title, author list, affiliations or correspondence in the supplementary files.

2 Supplementary Figures and Tables

For more information on Supplementary Material and for details on the different file types accepted, please see here. Figures, tables, and images will be published under a Creative Commons CC-BY licence and permission must be obtained for use of copyrighted material from other sources (including re-published/adapted/modified/partial figures and images from the internet). It is the responsibility of the authors to acquire the licenses, to follow any citation instructions requested by third-party rights holders, and cover any supplementary charges.

2.1 Supplementary Figures

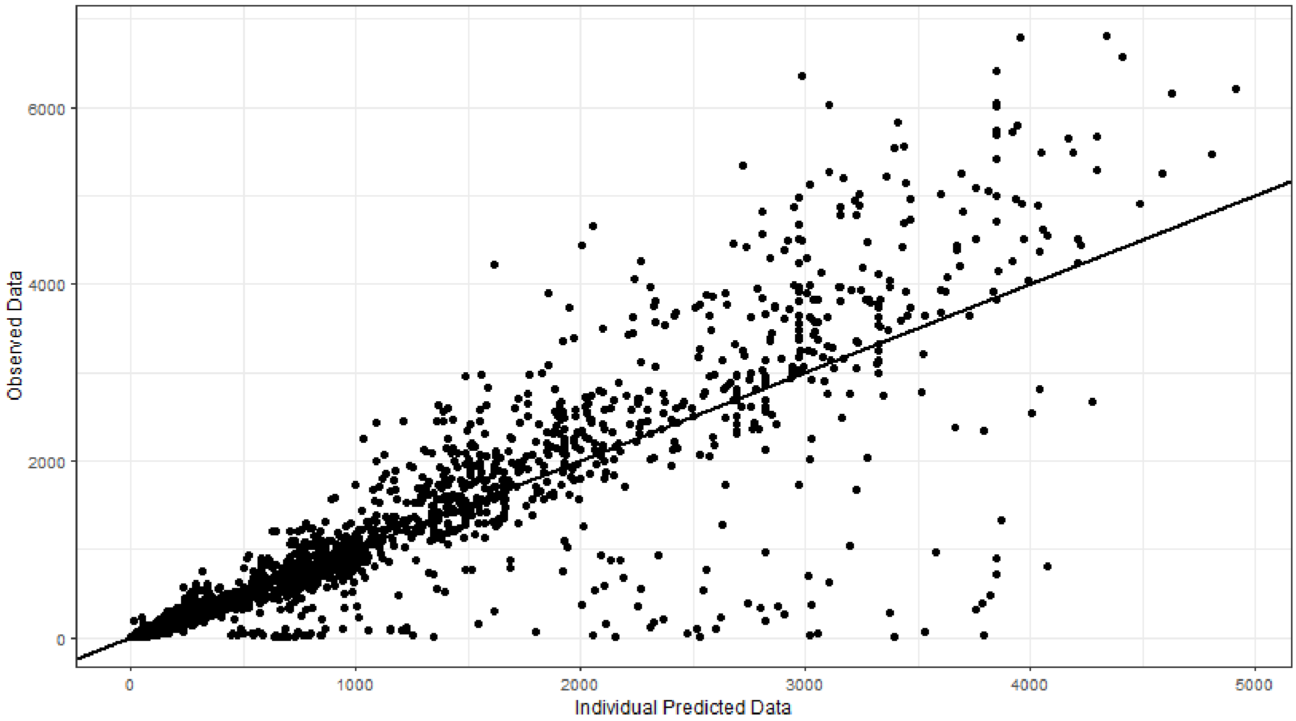

Figure S1: Model diagnostic plots of the final PK model for plasma torasemide concentration. Observed data versus individually predicted data (points) with a line of identity (black line) (linear and log scales).

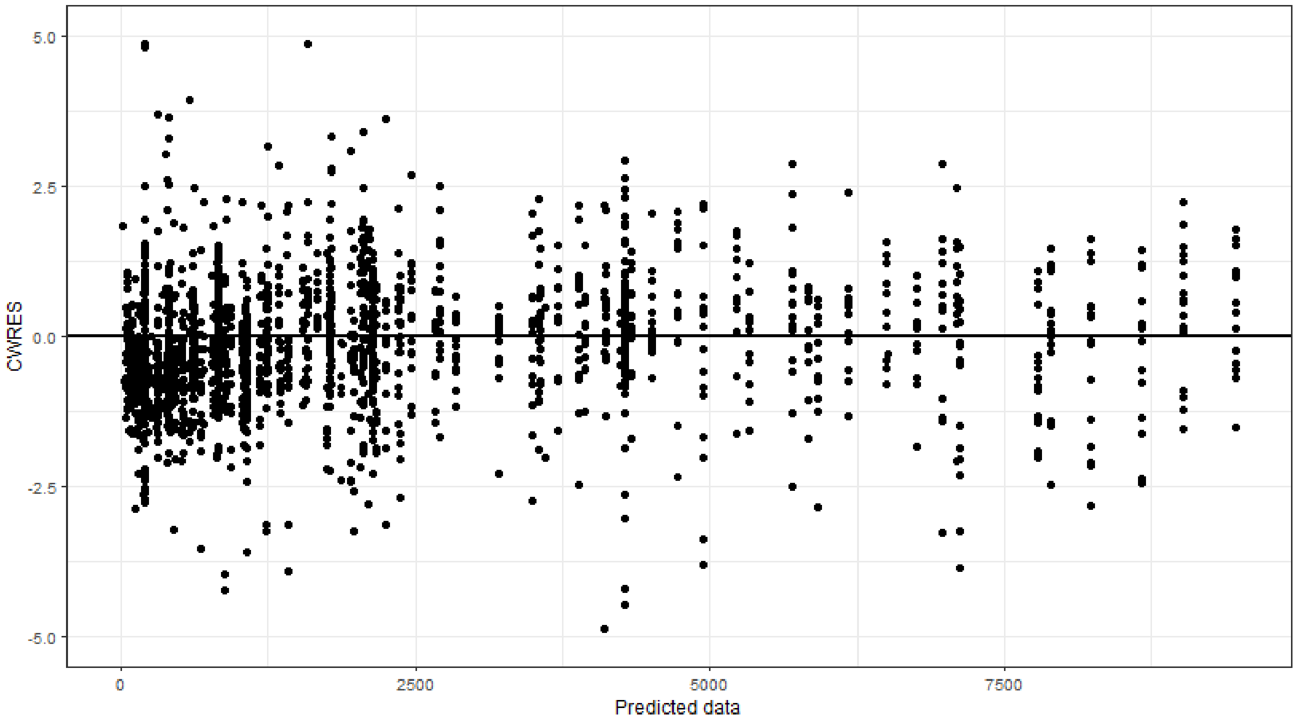

Figure S2: Model diagnostic plots of the final PK model for plasma torasemide concentrations. Conditional Weighted residuals (CWRES) versus Predicted data (point) with a zero line (black line)

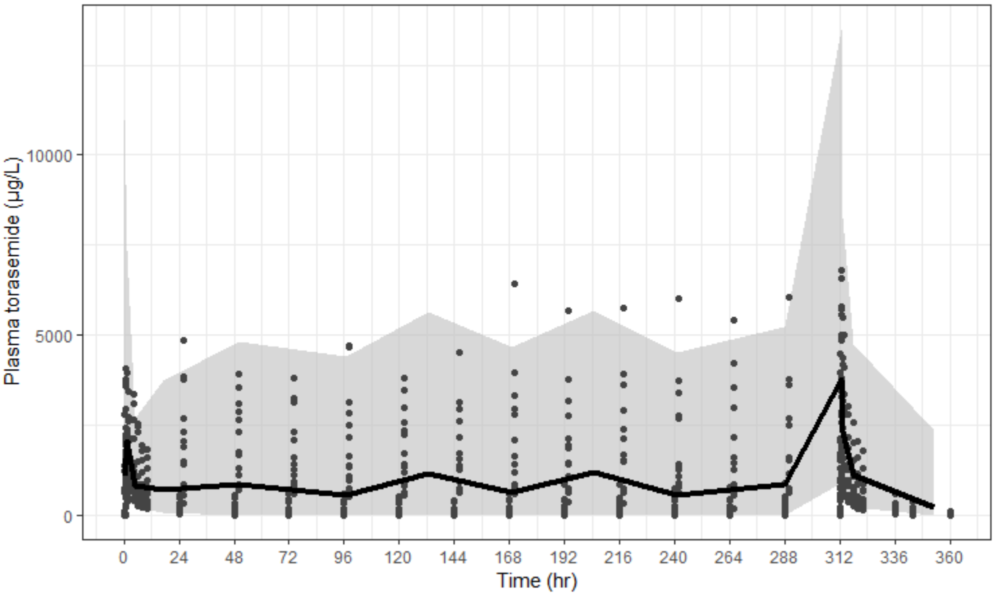

Figure S3: Visual Predictive Check of plasma concentration of torasemide following oral administration once a day of torasemide at 0.1, 0.2 and 0.4 mg/kg/day in dogs obtained in study 1 and 90% prediction interval obtained with the PK analysis (bold line is predicted median and grey area included between 5th and 95th quantiles).

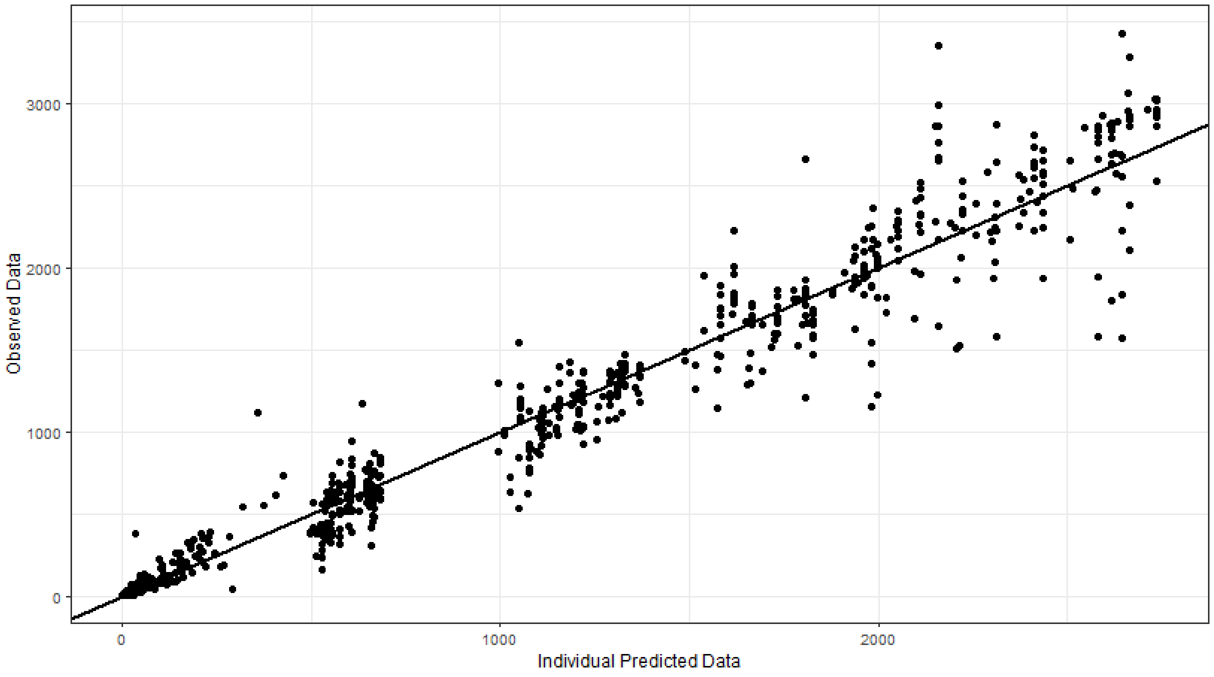

Figure S4 : Model diagnostic plots of the final PK model for torasemide quantity in urine. Observed data versus individually predicted data (points) with a line of identity (black line) (linear and log scales).

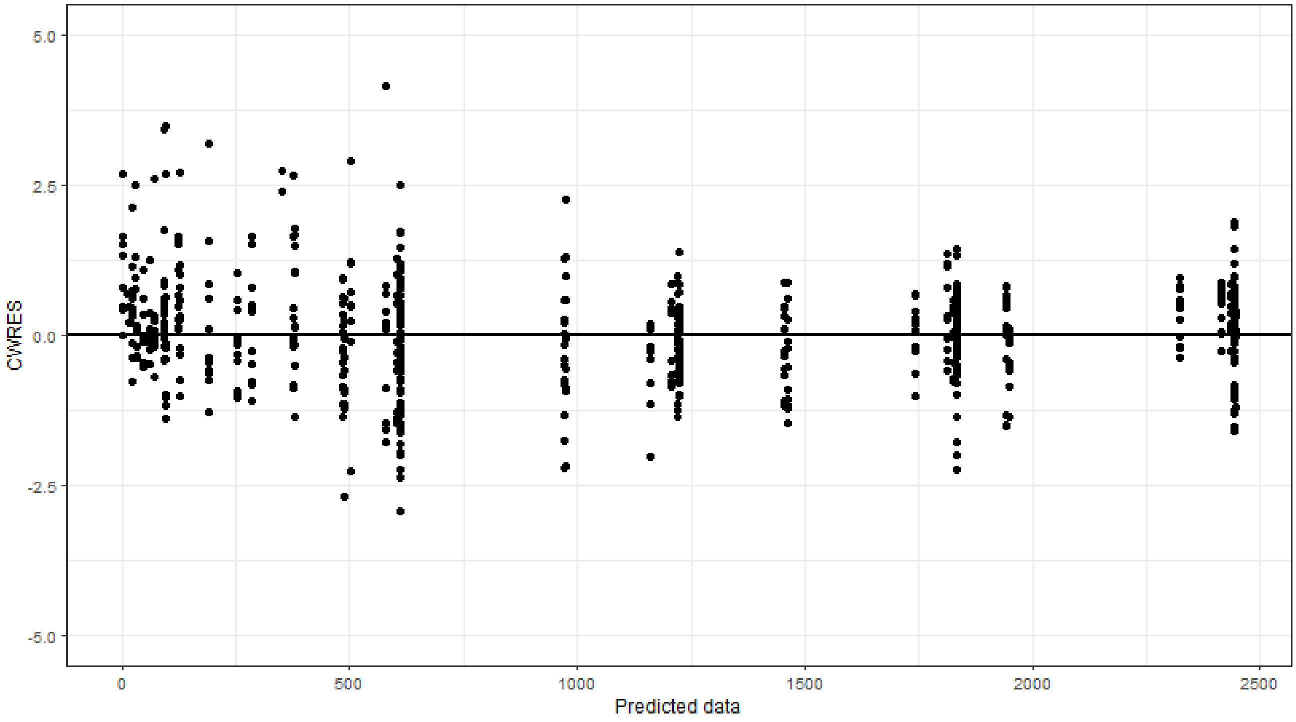

Figure S5: Model diagnostic plots of the final PK model for torasemide quantity in urine.

Conditional Weighted residuals (CWRES) versus Predicted data (point) with a zero line (black line)

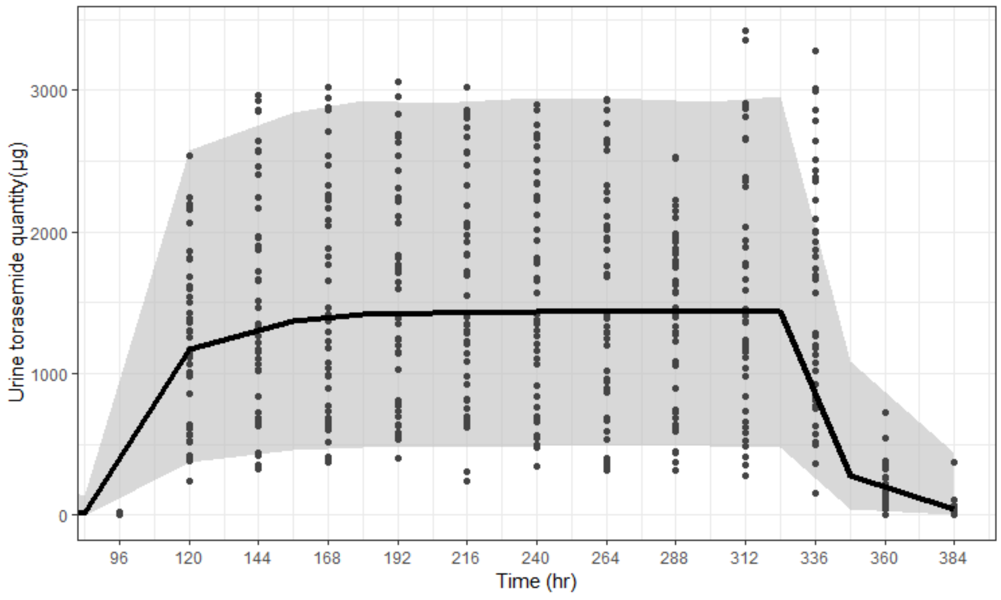

Figure S6: VPC of quantities of torasemide in urine following repeated oral administration once a day of C678 at 0.1, 0.2, 0.3 and 0.4 mg/kg/day in dogs obtained in study 2 and 90% prediction interval obtained with the PK analysis (bold line is predicted median and grey area included between 5th and 95th quantiles).

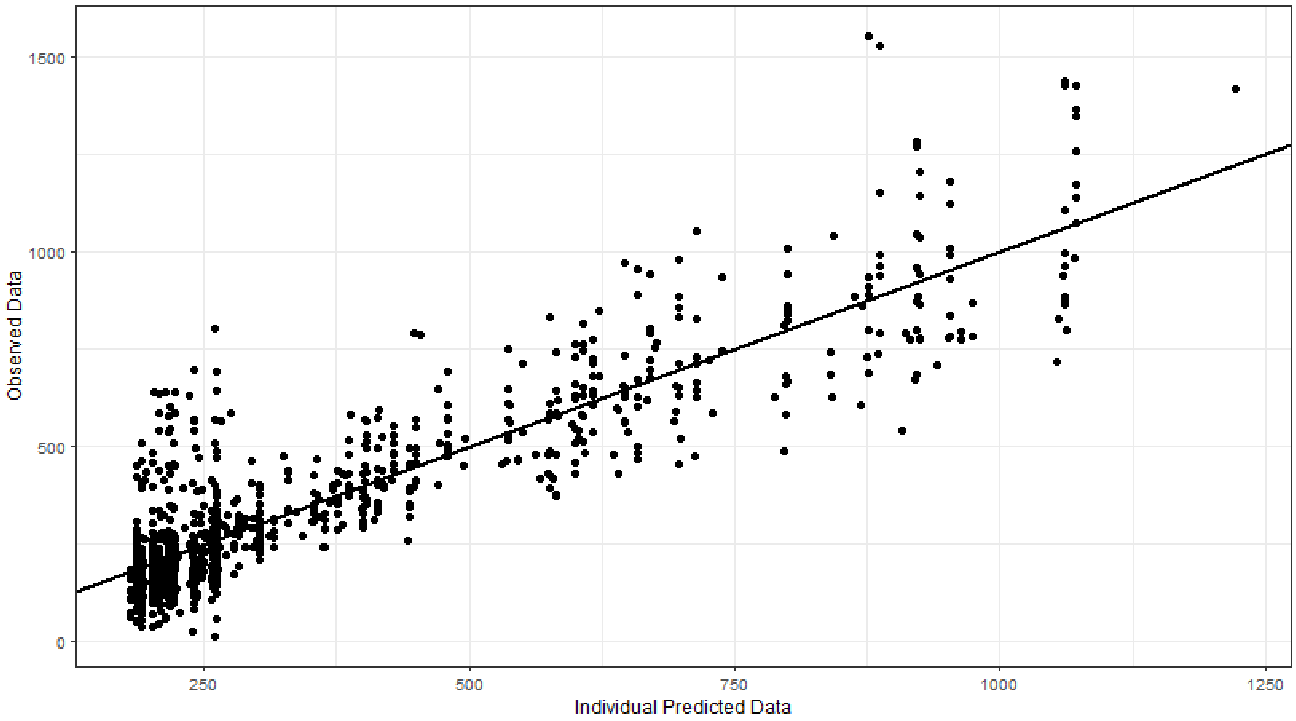

Figure S7: Model diagnostic plots of the final PD model on diuresis. Observed data versus individually predicted data (points) with a line of identity (black line)

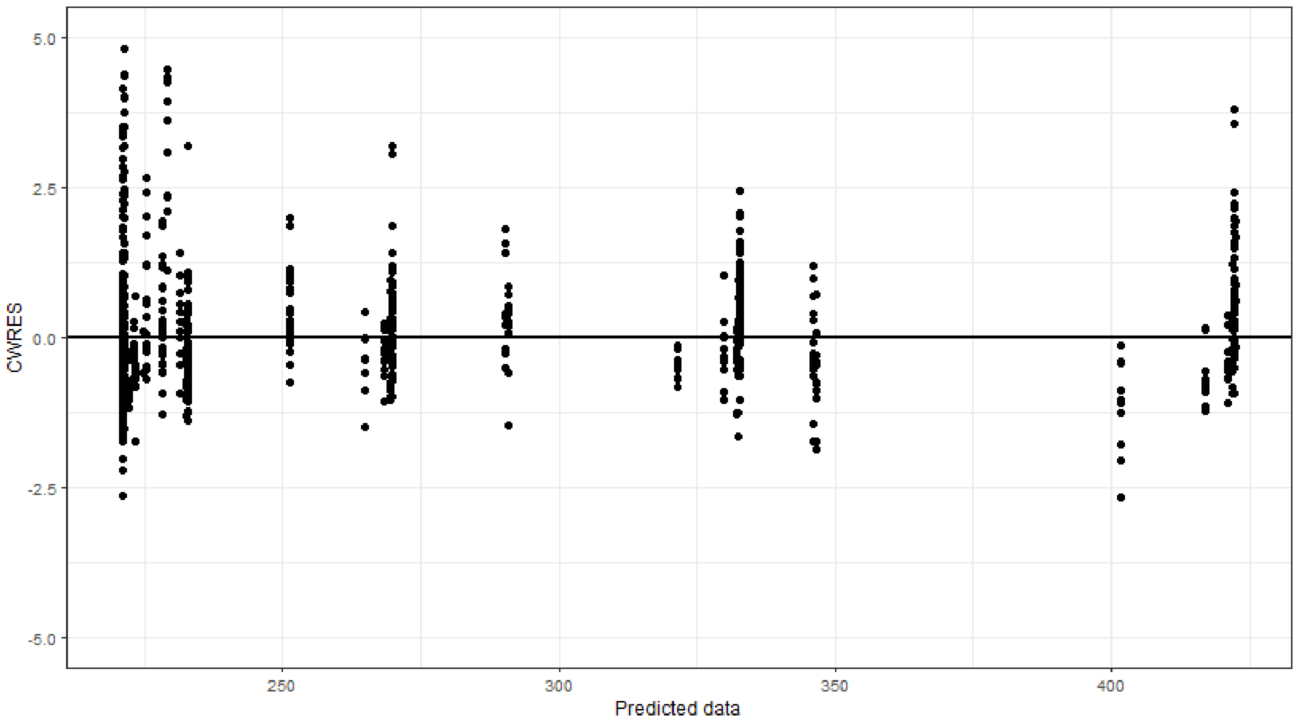

Figure S8: Model diagnostic plots of the final PD model on diuresis. Conditional Weighted residuals (CWRES) versus Predicted data (point) with a zero line (black line)

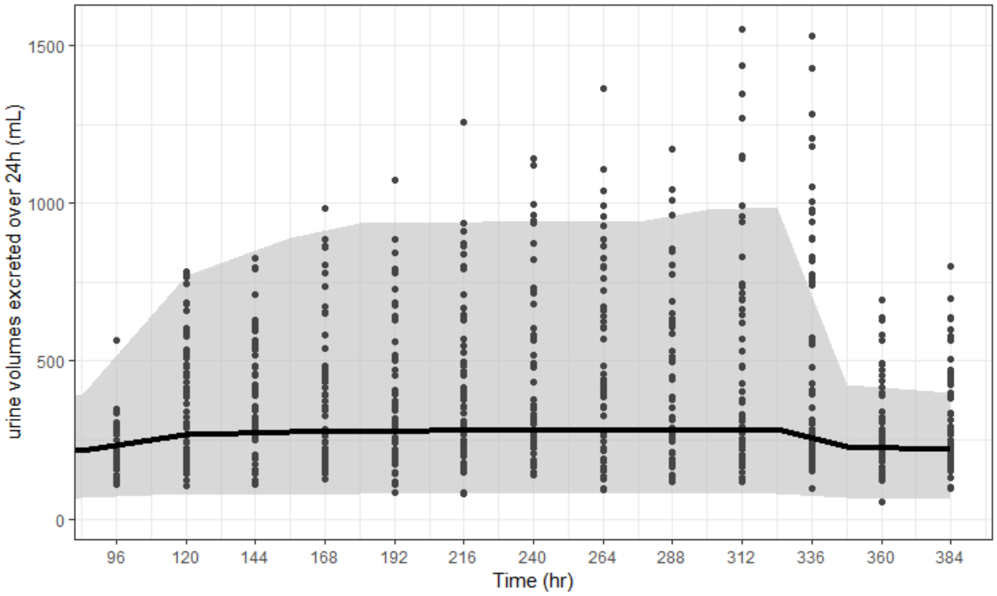

Figure S9: VPC of urine volumes excreted over 24 h profiles obtained after daily repeated administrations of 0.1, 0.2, 0.3 or 0.4 mg/kg/day of torasemide in dogs in study 2 and 90% prediction interval obtained with the PD analysis (bold line is predicted median and grey area included between 5th and 95th quantiles).

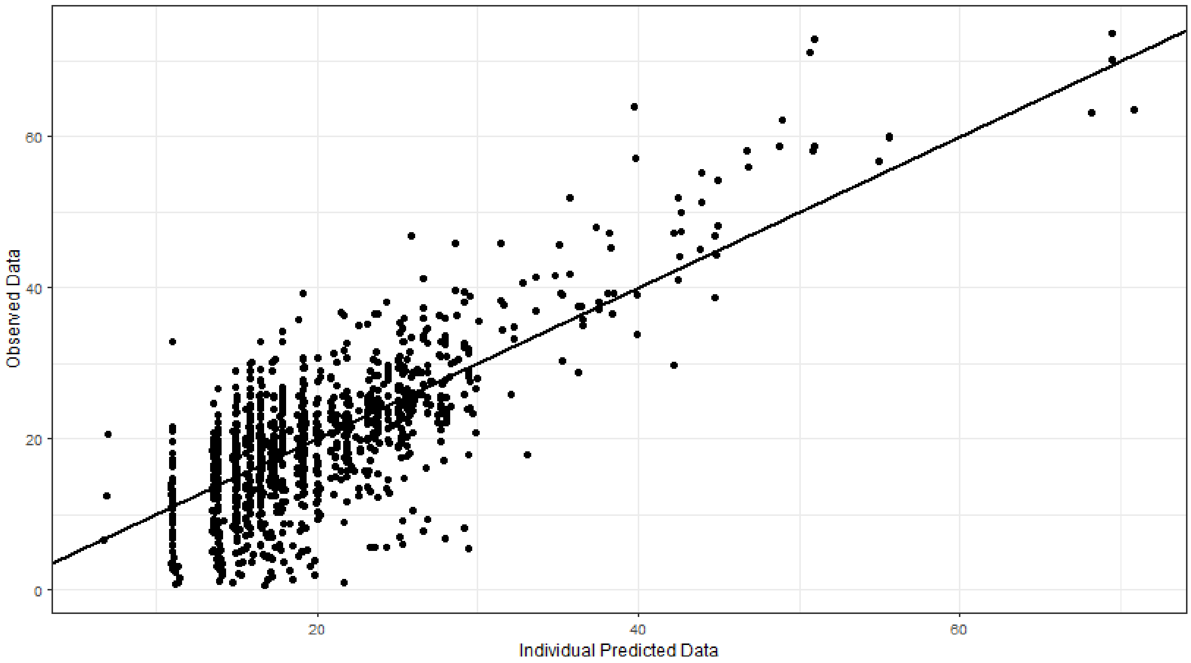

Figure S10: Model diagnostic plots of the final PD model on natriuresis. Observed data versus individually predicted data (points) with a line of identity (black line)

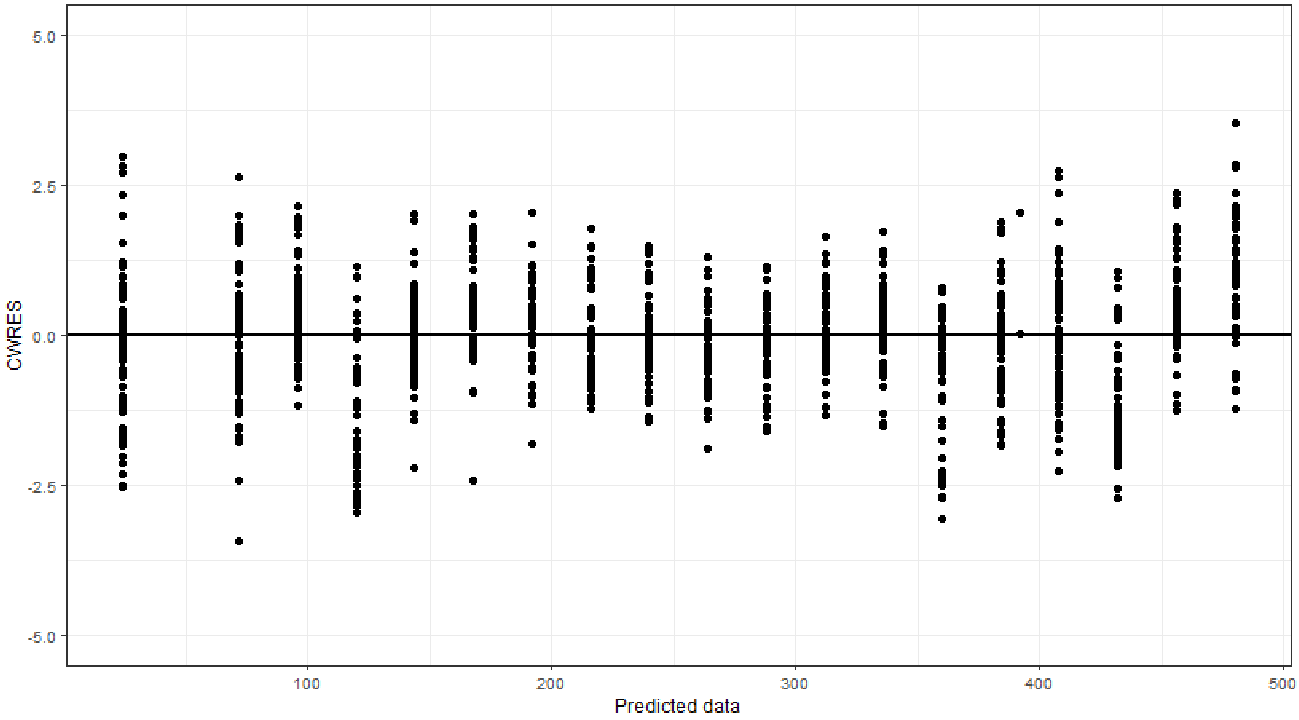

Figure S11: Model diagnostic plots of the final PD model on natriuresis. Conditional Weighted residuals (CWRES) versus Time (point) with a zero line (black line)

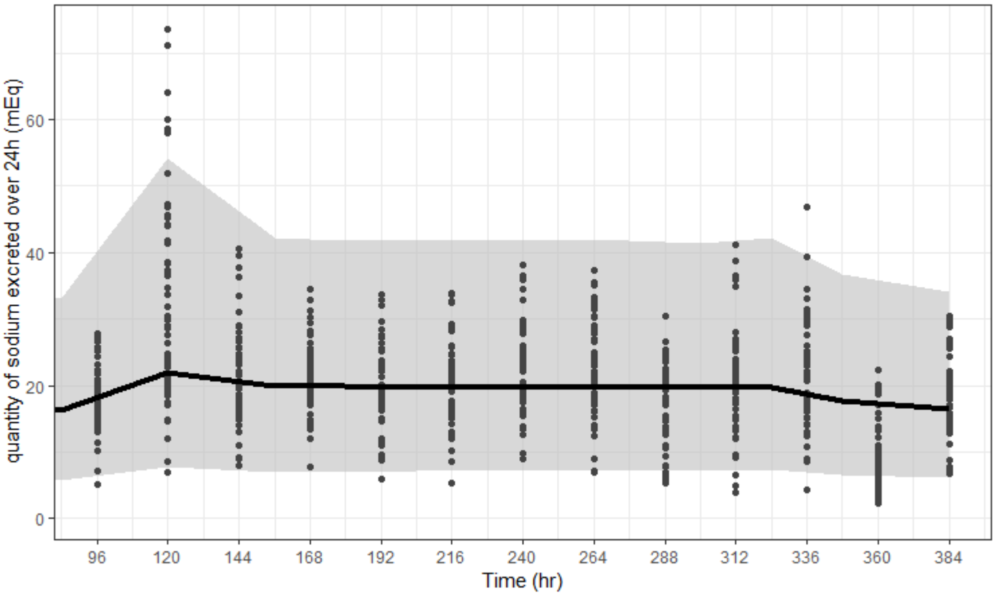

Figure S12: VPC of quantity of sodium excreted over 24 h profiles obtained after repeated administrations of 0.1, 0.2, 0.3 or 0.4 mg/kg/day of torasemide in dogs in study 2 and 90% prediction interval obtained with the PK analysis (bold line is predicted median and grey area included between 5th and 95th quantiles).

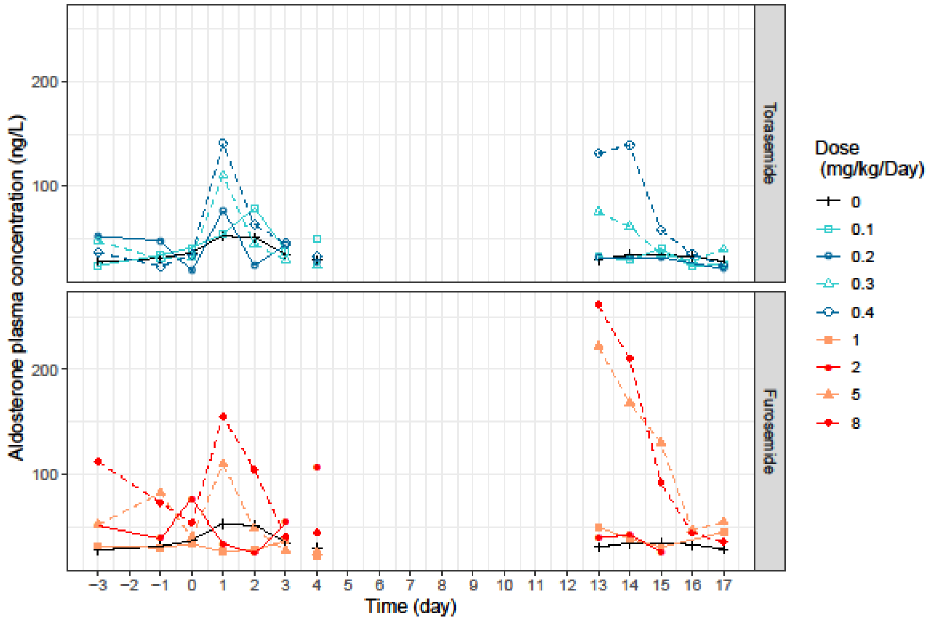

Figure S13: Mean serum aldosterone concentration after single or daily repeated oral administrations of 0.1, 0.2, 0.3, 0.4 mg/kg/day of torasemide once a day or 1, 2, 5 or 8 mg/kg/day of furosemide (in 2 daily administrations) in dogs of study 2.

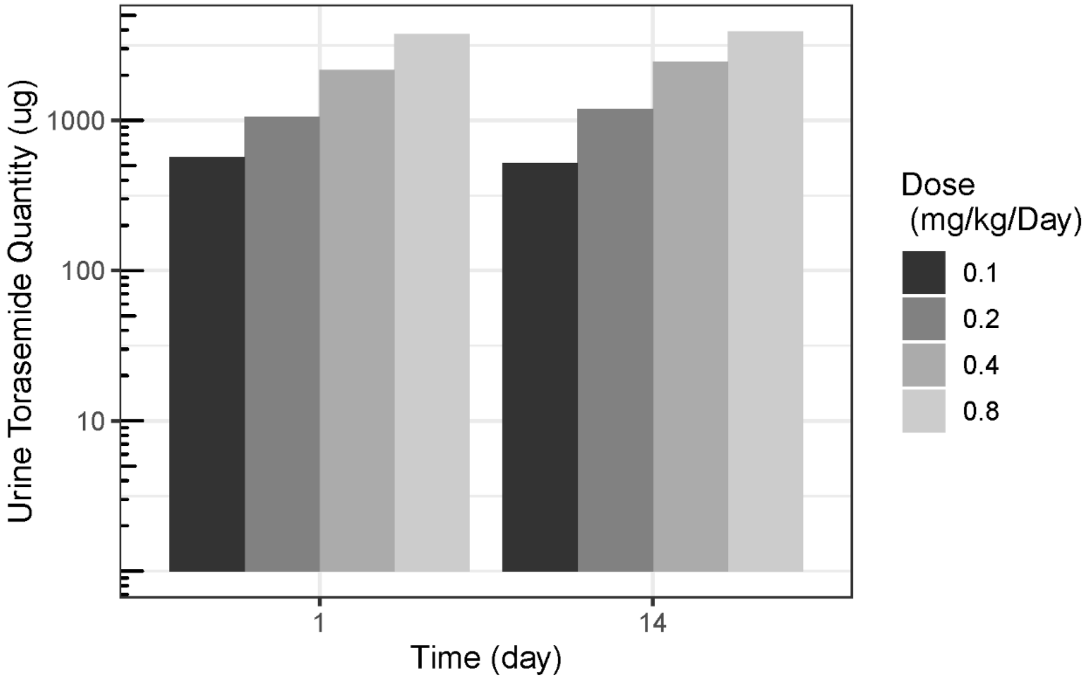

Figure S14: Torasemide urine quantity-time curve after daily administration for 14 days at doses of 0.1, 0.2, 0.4 and 0.8 mg/kg (Study 1)

2.2 Supplementary table

Table S2: Results from the validation of the bioanalytical method for torasemide in plasma and urine

			**Torasemide plasma (µg/L)**						**Torasemide urine (µg/L)**				
		**Concentrations evaluated (ng mL^{-1})**	**LLOQ**	**LQC**	**MQC**	**HQC**	**ULOQ**		**LLOQ**	**LQC**	**MQC**	**HQC**	**ULOQ**
			**5**	**10**	**150**	**3400**	**4000**		**20**	**40**	**600**	**10200**	**12000**
**Precision**		**Within run**	4.8	4.9	3.4	2.2	3.8		2.7	6.4	1.4	2.0	2.4
**(CV%)**		**Between-run (3 days)**	9.0	4.2	2.4	2.6	2.7		3.6	4.8	2.6	2.8	4.3
**Accuracy**		**Within run**	3.2	3.9	0.8	-2.6	1.7		5.7	3.1	4.0	4.5	8.0
**(RSE%)**		**Between-run (3 days)**	1.7	3.2	1.1	-1.8	1.3		4.4	2.4	1.5	1.6	2.8
**Calibration curve**		**Equation**											
**(Linearity)**		**Correlation coefficient**	**0.9982** (SD 0.001132)						**0.9988** (SD 0.000749)				
Abbreviations include: lower limit of quantification (LLOQ), low quality control (LQC), mean quality control (MQC), high quality control (HQC), upper limit of quantification (ULOQ), relative standard error (RSE) and coefficient of variation (CV).													
